# 2-Aminobenzoxazole-appended coumarins as potent and selective inhibitors of tumour-associated carbonic anhydrases

**DOI:** 10.1080/14756366.2021.1998026

**Published:** 2021-12-11

**Authors:** Alma Fuentes-Aguilar, Penélope Merino-Montiel, Sara Montiel-Smith, Socorro Meza-Reyes, José Luis Vega-Báez, Adrián Puerta, Miguel X. Fernandes, José M. Padrón, Andrea Petreni, Alessio Nocentini, Claudiu T. Supuran, Óscar López, José G. Fernández-Bolaños

**Affiliations:** a Facultad de Ciencias Químicas, Ciudad Universitaria, Benemérita Universidad Autónoma de Puebla, Puebla, México; b BioLab, Instituto Universitario de Bio-Orgánica “Antonio González” (IUBO-AG), Universidad de La Laguna, La Laguna, Spain; c NEUROFARBA Department, Sezione di Scienze Farmaceutiche e Nutraceutiche, University of Florence, Florence, Italy; d Departamento de Química Orgánica, Facultad de Química, Universidad de Sevilla, Seville, Spain

**Keywords:** Carbonic anhydrases, coumarins, benzoxazoles, antiproliferative agents, docking

## Abstract

We have carried out the design, synthesis, and evaluation of a small library of 2-aminobenzoxazole-appended coumarins as novel inhibitors of tumour-related CAs IX and XII. Substituents on C-3 and/or C-4 positions of the coumarin scaffold, and on the benzoxazole moiety, together with the length of the linker connecting both units were modified to obtain useful structure-activity relationships. CA inhibition studies revealed a good selectivity towards tumour-associated CAs IX and XII (*K*_i_ within the mid-nanomolar range in most of the cases) in comparison with CAs I, II, IV, and VII (*K*_i_ > 10 µM); CA IX was found to be slightly more sensitive towards structural changes. Docking calculations suggested that the coumarin scaffold might act as a prodrug, binding to the CAs in its hydrolysed form, which is in turn obtained due to the esterase activity of CAs. An increase of the tether length and of the substituents steric hindrance was found to be detrimental to *in vitro* antiproliferative activities. Incorporation of a chlorine atom on C-3 of the coumarin moiety achieved the strongest antiproliferative agent, with activities within the low micromolar range for the panel of tumour cell lines tested.

## Graphical Abstract

**Figure:**
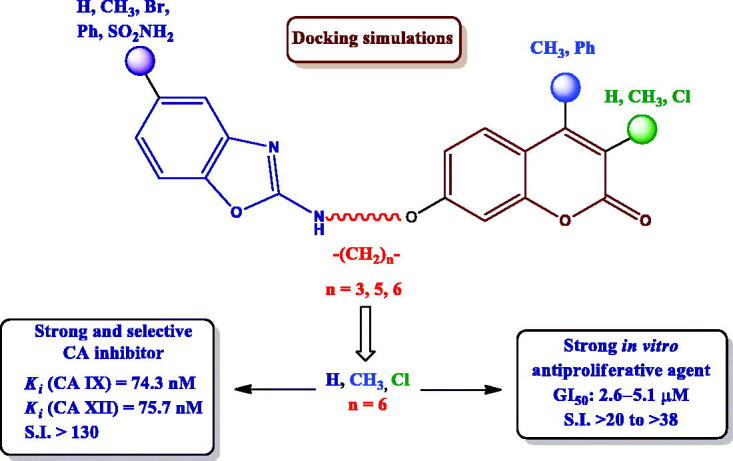

## 1. Introduction

Carbonic anhydrases (CAs, EC 4.2.1.1) are ubiquitous (Metallo)enzymes distributed across all life kingdoms and encoded into eight genetic families^1^: *α*-(primarily invertebrates, but also in protozoa, algae, the cytoplasm of green plants, and numerous Gram-negative bacteria), *β*- (bacteria, fungi, algae, some archea, and chloroplasts of mono-and di-cotyledons), *γ*-(most types of bacteria), *δ*-, *ζ-*(marine diatoms)*, η-*(protozoa), *θ*-(marine diatoms), *ι-*(marine phytoplankton and bacteria), the last one just recently discovered^2^. In turn, mammalian *α*-CAs are categorised into 16 isoforms, classified according to their tissue distribution and kinetic properties^3^: cytosolic (CA I, II, III, VII, XIII), mitochondrial (CA VA, VB), membrane-bound (CA IV, IX, XII, XIV, XV), secreted from saliva and colostrum (CA VI) and CA-related proteins (CARP)^4^, which are catalytically inactive (CA VIII, X, XI).

The biological role of these enzymes is to catalyse the reversible hydration of CO_2_ to furnish bicarbonate and a proton; this simple process, which is too slow under physiological conditions to meet metabolic requirements^1^, was found to be essential for many biological events, like respiration (by dissolving CO_2_ in blood as HCO_3_^-^)^5^, maintenance of pH homeostasis^6^, ureagenesis or gluconeogenesis^7^. From a pharmacological point of view, a plethora of therapeutic involvements of CAs have been reported, in connection with glaucoma^8^, epilepsy^9^, neuropathic pain^10^, ischaemia^11^, obesity^12^, cancer^13^, and more recently, neurodegenerative disorders, like Alzheimer’s disease^14^. Therefore, the development of CA inhibitors^15^ and activators^16^ is a research area with an increasing interest in the medicinal chemistry area.

Although some metals, e.g. Cd(II), Co(II), Fe(II), Mn(II), have been identified as prosthetic groups in the active site of CAs, the most frequent one is Zn(II)^17^; it has been demonstrated that metal coordination geometries, together with their capacity to orchestrate the dynamics of the surrounding water network through long-range electrostatic effects, can modulate the catalytic efficiency^18^.

The most common family of CA inhibitors is comprised of sulphonamides and their isosteres (sulfamates, sulfamides), which behave as strong inhibitors by chelating the Zn^2+^ ion in the active site^17^; nevertheless, they are frequently endowed with moderate selectivity, which leads to a series of side-effects. Consequently, the search for alternative chemotypes of CA inhibitors is a hot topic nowadays. In this context, coumarins (2*H*-chromen-2-ones), which are abundant phytochemicals^19^, but also present in bacteria and fungi (more than 1300 natural structures have been identified so far) emerged as an interesting new family of CA inhibitors^20^.

Coumarins are considered as a privileged structure in medicinal chemistry^21^, exhibiting a plethora of bioactivities^22^, such as antioxidant^23^, anti-inflammatory^24^, antimicrobial^25^, anti-Alzheimer’s^26^^,^^27^, or antiproliferative^28^^,^^29^ properties. Conjugation of coumarins with a second pharmacophore is currently gaining attention to access multitarget drugs^30^. Many of such activities are the result of the inhibition of key enzymes by coumarin-containing derivatives^20^^,^^31–38^, either natural or synthetic; this is due to their peculiar planar structure and to the possibility of establishing strong non-covalent interactions involving the lactone moiety (hydrogen bonding, dipole-dipole) and the aromatic scaffold (π-π and cation-π interactions)^21^. Regarding CAs, the slow inhibition mode observed for coumarins compared to sulfonamido-derivatives suggested that they might behave as suicide inhibitors^39^. Kinetic, crystallographic and computational data revealed that coumarins act in fact as prodrugs^40^^,^^41^: they undergo hydrolysis on their lactone functionality by the esterase activity of the CAs, and the corresponding 2-hydroxycinnamic acids occlude the entrance to the enzyme active site. In particular, coumarin derivatives usually behave as selective inhibitors of CAs IX and XII, which are upregulated in several hypoxic tumours^3^^,^^42^, and are responsible for the acidic microenvironment in tumour cells. hCA IX expression is limited in normal tissues and is considered to be a marker of aggressive and resistant tumours^43^. Regarding hCA XII, its inhibition has been associated with the inactivation of the P-gp machinery, one of the mechanisms for eliminating xenobiotics, and therefore, correlated with the development of resistance towards chemotherapeutic drugs^44^.

**Figure 1.**
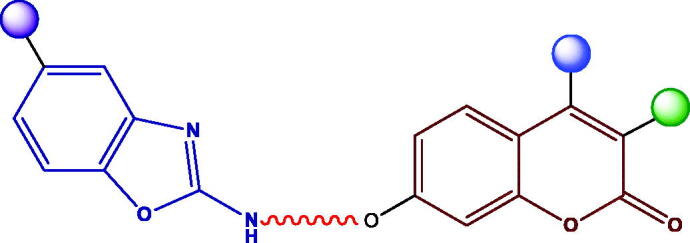
The general structure of the coumarin-benzoxazole hybrids is prepared herein.

We envisioned the preparation of a small library of the hitherto unknown coumarin-benzoxazole hybrids depicted in Figure 1 to develop novel inhibitors of hCAs IX and XII. The numerous pharmacological properties associated with the benzoxazole skeleton, together with the reduced toxicities of its derivatives^45^, stimulated us to incorporate such scaffold and analyse the possibility of interactions with CAs.

The structure of these novel hybrids is comprised of three key structural motifs: the coumarin skeleton, acting as a prodrug against CAs, and decorated with different substituents on C-3 and C-4 positions; the 2-aminobenzoxazole scaffold, that might establish non-covalent interactions with both, the hydrophobic and the hydrophilic regions of the enzyme (π-π interactions, hydrogen bonding); and the linker, a hydrocarbon chain with different lengths, providing conformational flexibility to the heterocyclic residues.

## 2. Materials and methods

### 2.1. General procedures

TLCs (Merck 60 F_254_, gel thickness 0.25 mm) were performed using aluminium-coated sheets, using the eluant indicated in the experimental section. Spots were visualised by UV light (*λ* = 254 nm), and by charring with 10% ethanolic vainillin containing 1% H_2_SO_4_, or with 5% ethanolic phosphomolybdic acid.

Column chromatography purifications were performed using silica gel stationary phase (Merck 60, particle size 40‒63 µm), eluting by gravity, or with mild pressure, using the eluant indicated in the experimental section.

NMR spectra were registered in the Centro de Investigación, Tecnología e Innovación de la Universidad de Sevilla (CITIUS), using Bruker Avance III 300 and 500 spectrometers (300 and 500 MHz for ^1^H, 75.5 and 125.7 MHz for ^13 ^C), and the deuterated solvents indicated in each case. Chemical shifts (*δ*) are expressed in ppm, and coupling constants (*J*), in Hz. Residual signals from the solvent are used as internal references^46^. Mass spectra were registered using a Qexactive spectrometer, using Electrospray Ionisation (ESI).

### 2.2. Chemistry

#### 2.2.1. General procedure for the preparation of azides 5a–f

To a solution of the corresponding ω-bromoderivative **4a***–***f** (1.0 equiv.) in DMF (5 ml) was added NaN_3_ (3.0 equiv.), and the corresponding mixture was heated at 70 °C for 3–4 h. After that, it was extracted with EtOAc (3 × 30 ml); the organic layer was washed with brine (3 × 20 ml), H_2_O (3 × 20 ml), dried over Na_2_SO_4,_ and filtered. The filtrate was concentrated to dryness under reduced pressure to give **5a***–***f** in quantitative yields, which were used for the next step without any further purification.

#### 2.2.2. General procedure for the preparation of amines 6a–f

To a solution of **5a***–***f** (106–240 mg) in MeOH (5 ml) was added Pd(OH)_2_/C (10–20 mg). The resulting suspension was subjected to standard hydrogenolysis at rt and 1 atm H_2_ for 4 h. The catalyst was removed by filtration through a Celite® pad, and the filtrate was concentrated to dryness to give **6a**–**f**, which were obtained in quantitative yields and used directly for the next step without any further purification.

#### 2.2.3. General procedure for the preparation of isothiocyanates 7b,c,e,f

To a vigorously stirred solution of the amines **6b**,**c**,**e**,**f** (1.0 equiv.) in a 1:1 CH_2_Cl_2_/H_2_O mixture (20 ml) were added CaCO_3_ (3.0 equiv.) and thiophosgene (1.5 equiv.); the corresponding mixture was kept stirring for 30 min. Then, it was filtered through a Celite^®^ pad and the filtrate was extracted with CH_2_Cl_2_ (3 × 30 ml) and washed with brine (3 × 20 ml) and H_2_O (3 × 20 ml). The organic layer was dried over Na_2_SO_4_, filtered and the filtrate was concentrated to dryness to give **7b**,**c**,**e**,**f** which were used for the next step without any further purification.

#### 2.2.4. General procedure for the preparation of benzoxazoles 8a–j

Method A. To a solution of amines **6a**,**d** in CH_2_Cl_2_ (5 ml), thiocarbonyldiimidazole (TCDI) (1.5 equiv.) and DMAP (1.0 equiv.) were added; the corresponding mixture was stirred at rt and under Ar for 2 h. After that, it was concentrated to dryness under reduced pressure and redissolved in THF (5 ml); to this solution, the corresponding 2-aminophenol (1 equiv.) was added and it was refluxed for 17–40 h. Then, my mixture was allowed to cool down to rt, and TBAI (cat.) and 30% H_2_O_2_ (2.0 equiv.) were added; the mixture was stirred at rt for 1–2 h. After that, it was concentrated to dryness and the residue was purified by column chromatography (7:3 Cyclohexane–EtOAc) to give derivatives **8a**,**d** (See Supplementary Material).

Method B. To a solution of isothiocyanates **7b**,**c**,**e**,**f** in THF (5 ml) the corresponding 2-aminophenol (1.0 equiv.) was added and the mixture was refluxed for 17–40 h. After cooling down to rt, 30% H_2_O_2_ (2.0 equiv.) and TBAI (cat.) were added. This mixture was stirred at rt for 1–2 h; then, it was concentrated to dryness and the residue was purified by column chromatography (7:3 Cyclohexane–EtOAc) to give derivatives **8b**,**c**,**e**–**j** (See Supplementary Material).

### 2.3. CA inhibition assays

A stopped-flow CO_2_ hydrase assay has been employed as reported earlier^10–12^. All enzymes were recombinant proteins obtained in-house as reported^10–12^ and their concentrations in the assay system were in the range of 5–12 nM.

### 2.4. Antiproliferative assays

The antiproliferative assays were conducted following the protocol of the US National Cancer Institute (NCI), with minor modifications^47^.

### 2.5. Docking simulations

Structures for all proteins (CA IX: PDBid 5FL4; CA XII: PDBid 4HT2) were retrieved from the Protein DataBank^48^. Crystal structures were optimised using the QuickPrep protocol from MOE (Chemical Computing Group). All ligands were drawn, hydrogens added, and geometry optimised with MOE. For the docking calculations, performed with MOE, in the placement stage, we used the Triangle Matcher algorithm with the London dG scoring scheme. In the refinement stage, we kept the receptor rigid and used the GBVI/WSA dG scoring scheme.

## 3. Results and discussion

### 3.1. Chemistry

**Scheme 1.**
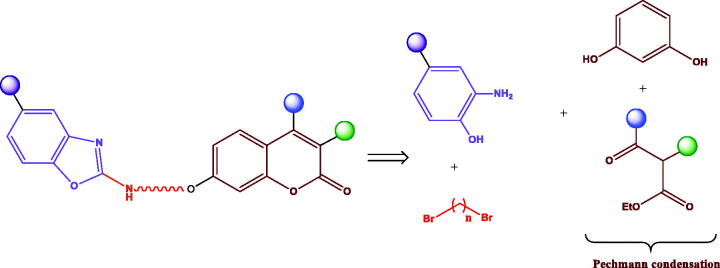
Retrosynthetic analysis for the preparation of coumarin-benzoxazole hybrids

The retrosynthetic analysis for accessing the coumarin-benzoxazole hybrids proposed herein is depicted in Scheme 1; the key starting materials for accessing such compounds are 4-substituted 2-aminophenols, resorcinol, β-ketoesters and α,ω-dibromoalkanes.

**Scheme 2.**
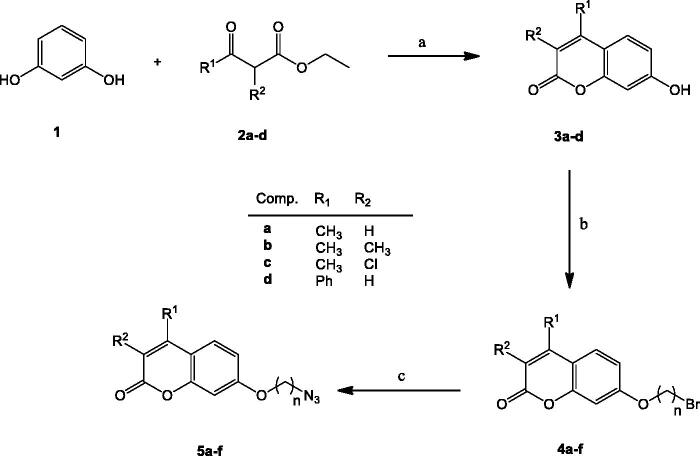
Preparation of ω-azidoalkyl derivatives **5a–f**. Reagents and conditions: (a) H_2_SO_4_, 0 °C→rt; (b) α,ω-Dibromoalkane, anyh. K_2_CO_3_, CH_3_CN, reflux; (c) NaN_3_, DMF, 70 °C.

The synthetic pathway started with the acid-catalysed Pechmann condensation^49^ involving resorcinol **1** and substituted β-ketoesters **2** to furnish umbelliferone derivatives **3a**–**d**, bearing different substituents on C-3 and/or C-4 positions of the coumarin moiety (Scheme 2). It has been reported that alkyl substitution on those positions decreases the potential hepatotoxicity of such derivatives, by decreasing the rate of the formation of a transient 3,4-epoxide moiety upon metabolisation^50^.

**Scheme 3.**
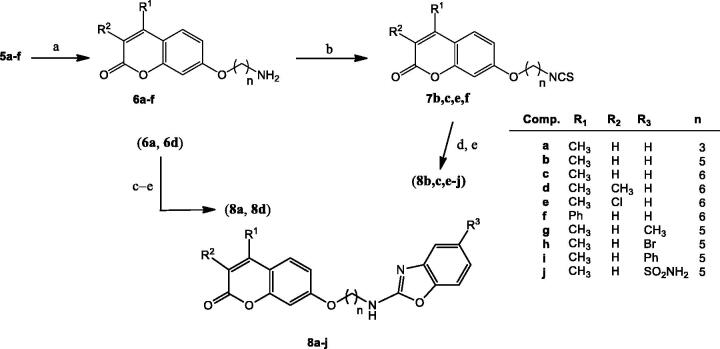
Preparation of coumarin-benzoxazole hybrids **8a–j**. Reagents and conditions: (a) H_2_, Pd(OH)_2_, MeOH; (b) CSCl_2_, CaCO_3_, 1:1 CH_2_Cl_2_−H_2_O, rt; (c) TCDI, DMAP, CH_2_Cl_2_, rt; (d) Corresponding *o*-aminophenol, TFH, reflux; (e) H_2_O_2_, TBAI, THF, rt.

Next, the hydroxyl group on C-7 position was subjected to a Williamson synthesis, using an excess of an α,ω-dibromoalkane to favour the monosubstitution process, under mild basic conditions (Scheme 2). Subsequent nucleophilic substitution with NaN_3_, followed by Pd-catalysed hydrogenolysis of the corresponding azido derivative **5a**–**f** afforded amino-alkyl counterparts **6a**–**f** (Scheme 3). Transformation of the amino moiety into the corresponding isothiocyanate (alternatively with TCDI or CSCl_2_), coupling with an *o*-aminophenol to furnish a transient and not isolated thiourea, and final H_2_O_2_/TBAI-promoted cyclodesulfurization^51^ furnished target benzoxazoles **8a**–**j** (Scheme 3).

TCDI was tentatively used as a green substitute for thiophosgene in the preparation of isothiocyanates. Attempts to isolate the corresponding isothiocyanate upon isothiocyanation reaction of **6a,d** gave rather modest yields (roughly 17%, Scheme 3); increase of the reaction times led to decomposition of the isothiocyanate. It was then assumed that reaction of TCDI with coumarin-amino derivatives **6a,d** was not complete, and presumably, a thioureido derivative involving the amino-coumarin and one of the imidazole units from TCDI was obtained as the major compound instead of the heterocumulenes **7**. Interestingly, the addition of 2-aminophenol to the crude reaction mixture gave the corresponding transient thiourea, as evidenced by TLC. Final *in situ* iodide-catalyzed oxidative cyclodesulfurization reaction by treatment of thioureas with H_2_O_2_ and a catalytic amount of TBAI (Scheme 3) allowed the isolation of benzoxazoles **8a** and **8d** in a 45% and 34% yield for the *one-pot* three-step procedure.

Access to benzoxazoles turned out to be more practical, with fewer side-products, when CSCl_2_ was used as the thionating agent, as amino derivatives **6b**,**c**,**e**,**f** could be transformed quantitatively into the expected isothiocyanates **7b**,**c**,**e**,**f** (Scheme 3), in a three-phase medium (H_2_O-CH_2_Cl_2_-CaCO_3_) and under mild conditions. Isothiocyanates were isolated from the crude reaction mixture just by liquid-liquid extraction, without the need for chromatographic purification. Transformation of the heterocumulenes into the transient and non-isolated thioureas upon coupling with substituted *o*-aminophenols, followed by *in situ* cyclodesulfurization reaction afforded target benzoxazoles **8b**,**c**,**e**–**j** in moderate to good yields (25–67%, two steps, Scheme 3). For derivatives **8h** and **8j**, a Fisher-Porter tube was required for the formation of the thioureas; reduced reactivity of the corresponding *o*-aminophenols might be due to the electron-withdrawing effects of the bromine and sulfonamido substituents.

Cyclodesulfurization was confirmed by ^1^H- and ^13 ^C-NMR; thus, resonances at 4.97‒5.95 (assigned to the NH proton), and at 157.6‒161.7 ppm (C = N) demonstrated the proposed structures. Moreover, spectra of compounds **8i** and **8j**, registered in CDCl_3_/CD_3_OD mixtures, evidenced the absence of the NH proton, due to chemical exchange with the solvent. The absence of a C = S moiety in ^13 ^C at roughly 180 ppm, confirmed the disappearance of the thioureido motif.

### 3.2. Biological assessments

#### 3.2.1. Carbonic anhydrase inhibition

The 10 new benzoxazole-coumarin hybrids prepared herein have been evaluated *in vitro* as potential inhibitors of therapeutically relevant hCAs using the stopped-flow CO_2_ hydration assay (Table 1) using the drug acetazolamide (AAZ) as control. Two different groups of such metalloenzymes have been used: cytosolic isoforms I (off-target), II (related to glaucoma^52^) and VII (involved in epilepsy and neuropathic pain^53^) and membrane-bound isoforms IV (involved in rheumatoid arthritis^54^), IX, XII (both of them overexpressed in hypoxic tumours^40^).

**Table 1.** Inhibition data (*K*_i_, nM) of compounds **8a–j** against human CAs I, II, IV, VII, IX, and XII^a^^,b^.

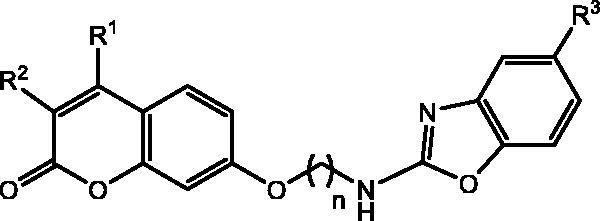								
Compound	CA I	CA II	CA IV	CA VII	CA IX	CA XII	Selectivity ratio I/IX // II/IX	Selectivity ratio I/XII // II/XII
**8a** (R^1^= CH_3_, R^2^=R^3^=H, *n* = 3)	>10,000	>10,000	839	>10,000	74.2	84.4	>134.8	>118.5
**8b** (R^1^= CH_3_, R^2^=R^3^=H, *n* = 5)	>10,000	>10,000	950	>10,000	70.7	84.5	>141.4	>118.3
**8c** (R^1^= CH_3_, R^2^=R^3^=H, *n* = 6)	>10,000	>10,000	984	>10,000	82.3	65.1	>121.5	>153.6
**8d** (R^1^=R^2^=CH_3_, R^3^=H, *n* = 6)	>10,000	>10,000	802	>10,000	64.5	69.8	>155.0	>143.3
**8e** (R^1^=CH_3_, R^2^=Cl, R^3^=H, *n* = 6)	>10,000	>10,000	820	>10,000	74.3	75.7	>134.6	>132.1
**8f** (R^1^=Ph, R^2^=R^3^=H, *n* = 6)	>10,000	>10,000	808	>10,000	271	316	>36.9	>31.6
**8g** (R^1^=R^3^=CH_3_, R^2^= H, *n* = 5)	>10,000	>10,000	762	>10,000	417	67.3	>24.0	>148.6
**8h** (R^1^= CH_3_, R^2^=H, R^3^= Br, *n* = 5)	>10,000	>10,000	792	>10,000	757	80.7	>13.2	>123.9
**8i** (R^1^= CH_3_, R^2^=H, R^3^= Ph, *n* = 5)	>10,000	>10,000	6265	>10,000	**33.2**	**57.1**	**>301.2**	**>175.1**
**8j** (R^1^= CH_3_, R^2^=H, R^3^= SONH_2_, *n* = 5)	806	516	3468	381	90.7	50.6	8.9 // 5.7	15.9 // 10.2
**AAZ**	250.0	12.0	74.0	2.5	25.0	5.7	10.0 // 0.48	43.9 // 2.1

^a^Mean from 3 different assays, by a stopped-flow technique (errors were in the range of ± 5–10% of the reported values); ^b^incubation time 6 h.

The following structure-activity relationships could be established:

- Compounds turned out to be inactive (*K*_i_ > 10,000 nM) against the cytosolic isoforms (I, II, VII), except for sulfonamido-containing **8j**, which exhibited submicromolar activities (*K*_i_= 806, 516 and 381 nM, respectively).
- Compounds **8a**–**h**, bearing no substituents on the phenyl residue of the benzoxazole moiety (R^3^= H), or small ones (R^3^= CH_3_, Br) exhibited moderate inhibition of hCA IV (*K*_i_= 762‒984 nM). On the contrary, bulky substituents and/or endowed with strong electron-withdrawing effects (R^3^= Ph, SO_2_NH_2_) led to weak inhibitors of this enzyme (**8i**, **8j**), with inhibition constants within the low micromolar range (6265 and 3468 nM, respectively).
- The linker length (*n* = 3, 5, 6) did not have a profound influence on the inhibition activities. Thus, a comparison of derivatives **8a**–**c** revealed a mild impairment on CA IV and IX inhibition and a moderate improvement on CA XII inhibition for the longest linker.
- Coumarin-benzoxazole hybrids behaved as selective inhibitors of tumour-associated hCAs IX and XII, with strong inhibitions (mid-nanomolar range) in most of the cases. In general, CA IX was more sensitive to the substitution pattern of the coumarin and the benzoxazole moieties.
- Disubstitution on the coumarin moiety (R^2^ position) with either a Me (**8d**) or a chlorine atom (**8e**) did not have a very appreciable influence. Nevertheless, incorporation of a Ph motif on R^1^ (monosubstituted derivative **8f**) led to a 3- and 5-fold impairment for the inhibition of CA IX and XII, respectively, compared to its Me-counterpart **8c**.
- Substitution of the benzoxazole moiety (R^3^) with CH_3_ and Br (derivatives **8g** and **8h**, respectively) was found to be detrimental for the activity against CA IX (6- and 11-fold decreased activity, submicromolar activities) when compared to their non-substituted counterpart **8b**.
- Substitution of the benzoxazole moiety (R^3^) with a Ph (**8i**) furnished strong inhibition of the tumour-associated CAs (*K*_i_ = 33.2 and 57.1 nM). The use of a sulfonamido motif in R^3^, despite providing the strongest CA XII inhibitor of the series, led to an outstanding loss of selectivity, due to the inhibition also of the off-target enzyme in the submicromolar range.

Comparison with native 4-methylumbelliferone **3a**^55^ (hCAI, hCAII > 100 µM; hCA IX 560 nM; hCAXII 8100 nM) revealed the outstanding increase in activity achieved with the hybrids reported herein (up to 16.9-fold for CA IX and up to 160.1-fold for CA XII).

#### 3.2.2. Antiproliferative activity

The coumarin-benzoxazole hybrids were also tested as antiproliferative agents against a panel of six human solid tumour cell lines (Table 2): A549 (non-small cell lung), HBL-100 (breast), HeLa (cervix), SW1573 (non-small cell lung) as examples of drug-sensitive lines, and T-47D (breast) and WiDr (colon) as multidrug-resistant lines. A non-tumour cell line (BJ-hTert, human fibroblasts) was also used for analysing the selectivity. Chemotherapeutic agents 5-fluorouracyl (5-FU) and cisplatin (CDDP) were included in the study as drug references.

**Table 2.** GI_50_ values (µM) for the antiproliferative activity of derivatives **8a–j.**

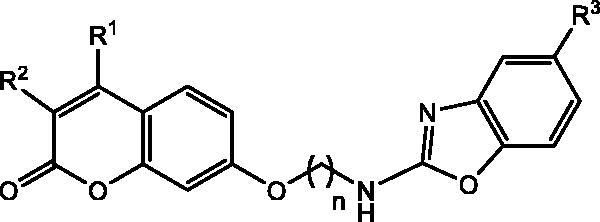							
Compound	A549 (Lung)	HBL-100 (Breast)	HeLa (Cervix)	SW1573 (Lung)	T-47D (Breast)	WiDr (Colon)	BJ-hTert
**8a** (R^1^= CH_3_, R^2^=R^3^=H, *n* = 3)	32 ± 1	41 ± 5	30 ± 2	29 ± 4	33 ± 3	37 ± 4	>50
**8b** (R^1^= CH_3_, R^2^=R^3^=H, *n* = 5)	15 ± 6	49 ± 6	39 ± 1	33 ± 4	38 ± 10	33 ± 1	>100
**8c** (R^1^= CH_3_, R^2^=R^3^=H, *n* = 6)	**8.3 ± 1.6**	>100	96 ± 7	>100	>100	>100	>100
**8d** (R^1^=R^2^=CH_3_, R^3^=H, *n* = 6)	15 ± 3	53 ± 14	29 ± 8	20 ± 3	>100	>100	>50
**8e** (R^1^=CH_3_, R^2^=Cl, R^3^=H, *n* = 6)	**2.6 ± 0.5**	**5.1 ± 1.4**	**3.9 ± 0.7**	**3.1 ± 0.5**	**4.3 ± 0.1**	**4.2 ± 0.5**	>100
**8f** (R^1^=Ph, R^2^=R^3^=H, *n* = 6)	>100	>100	>100	>100	>100	>100	>100
**8g** (R^1^=R^3^=CH_3_, R^2^= H, *n* = 5)	>100	>100	>100	>100	>100	>100	>100
**8h** (R^1^= CH_3_, R^2^=H, R^3^= Br, *n* = 5)	**6.5 ± 3.1**	23 ± 5	16 ± 1	14 ± 1	32 ± 7	29 ± 1	>100
**8i** (R^1^= CH_3_, R^2^=H, R^3^= Ph, *n* = 5)	41 ± 20	30 ± 9	28 ± 13	60 ± 4	>100	78 ± 38	25 ± 6
**8j** (R^1^= CH_3_, R^2^=H, R^3^= SONH_2_, *n* = 5)	13 ± 3	27 ± 8	21 ± 7	29 ± 8	34 ± 8	29 ± 9	>100
**5-Fluorouracil**	2.2 ± 0.3	4.4 ± 0.7	16 ± 5	3.3 ± 1.2	43 ± 16	49 ± 7	5.5 ± 0.5
**CDDP**	4.9 ± 0.2	1.9 ± 0.2	1.8 ± 0.5	2.7 ± 0.4	17 ± 3	26 ± 4	14 ± 2

Bold values emphasize the most active compounds

The following structure-activity relationships could be established:

- Substitution patterns had a deeper impact than in the inhibition assay.
- The order in potency as antiproliferative agents, considering substitution on the benzoxazole moiety (R^3^) was: **8h** > **8j** > **8b** > **8i** > **8g** (Br > SO_2_NH_2_ > H > Ph > CH_3_). Unexpectedly incorporation of a Me moiety (**8g**) completely abolished activity against all cell lines.
- Regarding substitution on the coumarin moiety, the use of a bulky substituent (R^1^=Ph, **8f**) completely abolished activity against all cell lines.
- Disubstitution with a second Me group (R^1^=R^2^=Me, **8d**) led to a clear impairment of activity against the multidrug-resistant cell lines in comparison with the monosubstituted counterpart (**8c**).
- Disubstitution with a chorine atom (R^1^= Me, R^2^=Cl) led to the strongest compound in the series (**8e**), with activities in the low micromolar range for all cell lines (GI_50_ = 2.6 − 5.1 µM); a remarkably increased activity was found for the multidrug-resistant cell lines compared to the chemotherapeutic agents included in the assay (up to 11.7-fold compared to 5-FU and up to 6.2-fold compared to CDDP).
- An increase in the tether length (compounds **8a**–**c**) provoked a strong impairment of activity; for line A549 this situation was completely reversed, leading to a strong antiproliferative activity for compound **8c** (GI_50_= 8.3 µM), the one with the longest linker.
- Regarding selectivity, most of the tested compounds lacked significant activity (GI_50_ > 100 µM, derivatives **8b,c,e–h,j**) against BJ-hTert cell line. Derivatives **8a** and **8d** exhibited weak activity against the non-tumour cell line; moreover, benzoxazole **8i** (R^1^=Me, R^3^=Ph) was a moderate antiproliferative agent against it, and thus the one with the poorest selectivity.
- Selectivity ranges of the lead compound of the series (**8e**, R^1^=Me, R^2^=Cl, > 19.6 – >38.5) clearly exceeded those found for the chemotherapeutic agents 5-FU (0.1‒2.5) and CDDP (0.5‒7.8).

#### 3.3.3. Docking studies

In order to get a deeper insight into the inhibition mechanism exerted by coumarin-benzoxazoles hybrids, compounds reported herein were subjected to docking studies with CAs IX and XII.

**Scheme 4.**
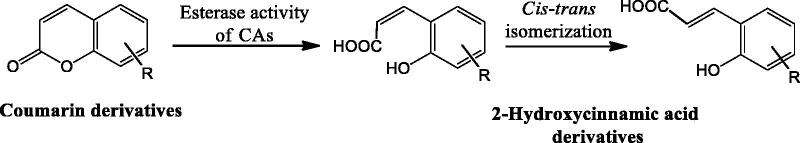
The mechanism for the inhibition of CAs by coumarins.

As aforementioned, CAs can also exert an esterase activity; the water molecule coordinated to the Zn^2+^ ion is activated by the metal, thus allowing it to act as a strong nucleophile^56^. Upon hydrolysis of the lactone functionality^39^, the corresponding 2-hydroxycinnamic acid might be isomerised to the most stable *E*-configuration, depending on the sterical hindrance of the substituents on the coumarin core (Scheme 4).

Compound **8i**, the strongest CA inhibitor within the series was taken as a model compound for the computational study. Firstly, energy minimisation between **8i**-CA complex was accomplished, considering both, the coumarin moiety (*closed form*) and its *E*-configured hydrolysed product (*open form*); data are depicted in Table 3. Such data are in agreement with previous reports^39^ that suggest the strongest interaction (lower docking interaction energies) of the hydrolysed structure with CAs IX and XII.

**Table 3.** Docking interaction energies of coumarin-benzoxazole hybrids **8i** (kcal/mol)

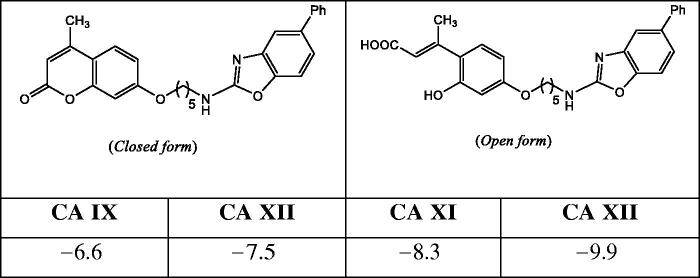			
CA IX	CA XII	CA XI	CA XII
−6.6	−7.5	−8.3	−9.9

Docking calculations of compound **8i** (*closed form*) complexed with CA IX showed H-arene interactions between Thr200 and the phenyl ring of the benzoxazole scaffold. Furthermore, van der Waals interactions with residues Asn65, Gly67, Gln92, His94, His96, Val121, Val130, Leu134, Val142, Thr201, and Pro203 were also observed. Interestingly, the *open form* of **8i**-CA IX complex revealed coordination of the carboxylate moiety with the Zn^2+^ ion of the catalytic site. This was also recently observed in molecular modelling of the interaction of psoralen derivatives and CAs^57^. Additionally, van der Waals interactions between **8i** *open form* and the residues Gln71, Gln92, His94, His96, Val121, Leu199, Th5200, Thr201, and Trp210 were also found.

**Figure 2.**
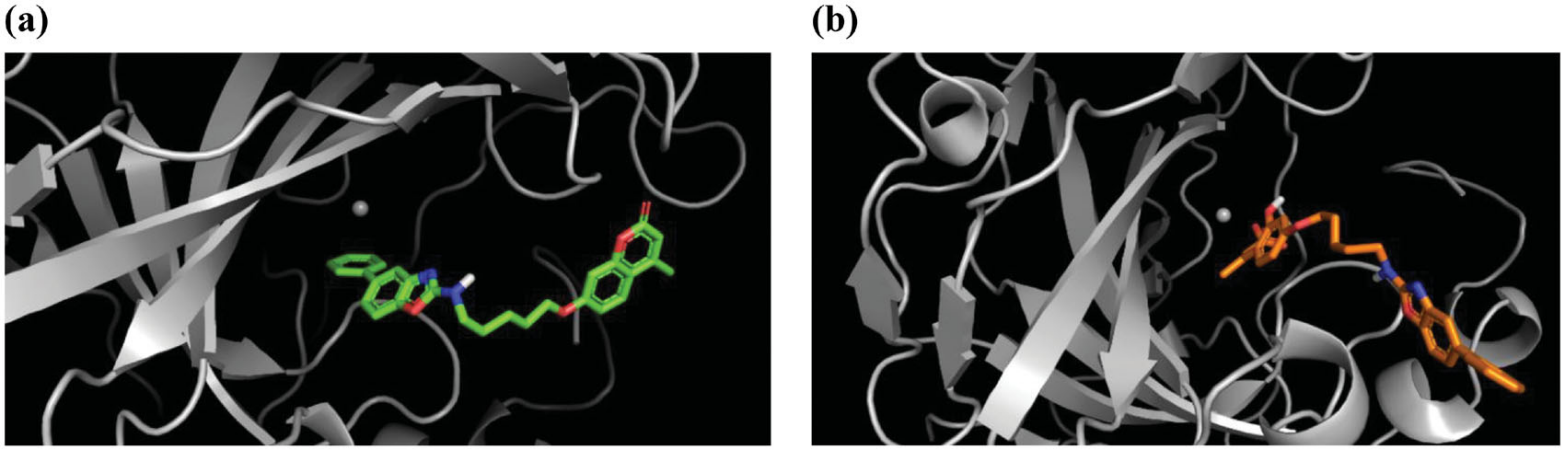
Predicted binding modes of the *closed-form* (a) and *open form* (b) of **8i** and CA IX.

Figure 2 shows the poses of both forms binding to CA IX, indicating a completely different orientation in both cases; while in the *closed-form* the benzoxazole moiety is directed towards the enzyme cleft (Figure 2(a)), the situation is reversed in the *open form* (Figure 2(b)), presumably due to the establishment of the strong ionic interaction between the deprotonated form of the cinnamic acid residue at physiological pH, and the Zn^2+^ cation.

**Figure 3.**
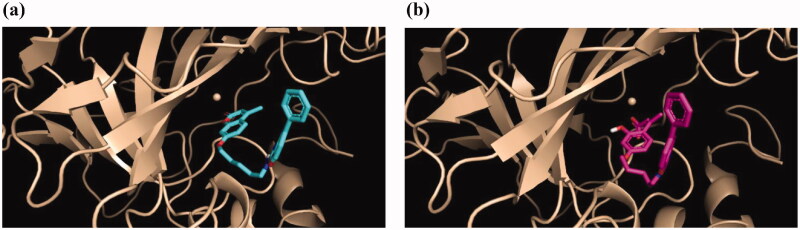
Predicted binding modes of the *closed-form* (a) and *open form* (b) of **8i** and CA XII.

A similar situation was found for the predicted interaction between **8i** and CA XII (Figure 3). In this case, π-π interactions between the *closed-form* of **8i** and His91 were detected. Furthermore, **8i** establishes van der Waals interactions with residues Asn64, Gln89, His93, His117, Val119, Ala129, Ser133, Leu139, Leu197, and Thr198. Docking calculations for the complexation of the *open form* of **8i** with CA XII also revealed coordination of the carboxylate moiety and the Zn^2+^ ion. Additionally, it established van der Waals interactions with residues Gln89, His91, His93, Glu104, His117, Val119, Ala129, Ser133, Leu139, Val141, Thr198, and Thr199. In this case, unlike CA IX in both structures, the coumarin scaffold is predicted to be directed to the enzyme cleft.

## 4. Conclusions

In conclusion, we herein report an unprecedented family of coumarin-2-aminobenzoxazole hybrids as selective inhibitors of tumour-associated hCAs IX and XII. Substituents on the coumarin and benzoxazole scaffolds, as well as the length of the tether connecting both of them, have been modified to obtain valuable structure-activity relationships. These compounds were accessed starting from substituted umbelliferones in a 6-step synthetic approach: monoalkylation on C-7 position with α,ω-dibromoalkanes, nucleophilic displacement with NaN_3_, hydrogenolysis, conversion of the terminal amino moiety into an isothiocyanate, coupling with *o*-aminophenols and intramolecular H_2_O_2_/tetrabutylammonium iodide (TBAI)-promoted cyclodesulfurization of the transient thioureas. CA inhibition studies revealed that most of title compounds behaved as strong and selective inhibitors of CAs IX and XII, with inhibition constants within the mid-nanomolar range. Coumarin-benzoxazole hybrids exhibited variable *in vitro* antiproliferative properties against a panel of human tumour cell lines, strongly dependent on the structural pattern. The lead compound (**8e**) exhibited GI_50_ values within the low micromolar range, with remarkable selectivities that exceeded the ones found for the control drugs. Therefore, the family of compounds described herein constitutes a promising start point for the future development of CA inhibitors as antiproliferative agents.

## Supplementary Material

Supplemental Material
