# Supplementary material for: 2-Aminobenzoxazole-appended coumarins as potent and selective inhibitors of tumour-associated carbonic anhydrases
Source: J Enzyme Inhib Med Chem. 2021 Dec 11;37(1):168–77. doi: 10.1080/14756366.2021.1998026 (PMC8667885; doi:10.1080/14756366.2021.1998026)
Supplement: Supplemental Material [file IENZ_A_1998026_SM6760.pdf]

2-Aminobenzoxazole-appended coumarins as potent and selective inhibitors of tumor-associated carbonic anhydrases

Alma Fuentes-Aguilar,[a] Penélope Merino-Montiel,[a] Sara Montiel-Smith,[a] Socorro Meza-Reyes,[a] José Luis Vega-Baez,*[,a] Adrián Puerta,[b] Miguel X. Fernandes,[b] José M. Padrón,*[,b] Andrea Petreni,[c] Alessio Nocentini,[c] Claudiu T. Supuran,*[,c] Óscar López,*[,d] José G. Fernández-Bolaños[d]

[a]*Facultad de Ciencias Químicas, Ciudad Universitaria, Benemérita Universidad Autónoma de Puebla, 72570, Puebla, PUE, México; e-mail: jose.vega@correo.buap.mx*

[b]*BioLab, Instituto Universitario de Bio-Orgánica "Antonio González" (IUBO-AG), Universidad de La Laguna, c/ Astrofísico Francisco Sánchez 2, E-38206 La Laguna, Spain; e-mail: jmpadron@ull.es*

[c]NEUROFARBA Department, Sezione di Scienze Farmaceutiche, University of Florence, 50019, Florence, Italy; *e-mail: claudiu.supuran@unifi.it*

[c]*Departamento de Química Orgánica, Facultad de Química, Universidad de Sevilla, Apartado 1203, E-41071 Seville, Spain. e-mail: osc-lopez@us.es*

Synthesis of benzoxazoles 8a-j...S2-S11

^{1}H- and ^{13}C-NMR spectra...S12-S31

S1. 7-[{3’-(Benzo[*d*]oxazol-2’’-ylamino)propyl}oxy]-4-methyl-2*H*-chromen-2-one (8a). Method A. Amino derivative **6b** (74.2 mg, 0.32 mmol, 1.0 equiv.), TCDI (85.0 mg, 0.48 mmol, 1.5 equiv.), DMAP (38.9 mg, 0.32 mmol, 1.0 equiv.), 2-aminophenol (34.7 mg, 0.32 mmol, 1.0 equiv.), 30% H_2O_2 (65 µL, 0.64 mmol, 2.0 equiv.) and TBAI (2.6%, 3 mg) were used. Yield: 51 mg (45%, 3 steps), brown foam. R_f 0.33 (1:1 Cyclohexane–EtOAc); ^{1}H-NMR (300 MHz, $CDCl_3$) δ 7.42 (d, 1H, $J_{5,6}$ = 8.8 Hz, H-5), 7.32 (d, 1H, $J_{H,H}$ = 7.7 Hz, Ar-H), 7.21 (d, 1H, $J_{H,H}$ = 7.7 Hz, Ar-H), 7.12 (td, 1H, $J_{H,H}$ = 7.6 Hz, $J_{H,H}$ = 0.9 Hz, Ar-H), 7.00 (td, 1H, $J_{H,H}$ = 7.9 Hz, $J_{H,H}$= 1.1 Hz, Ar-H), 6.82 (dd, 1H, $J_{5,6}$ = 8.8 Hz $J_{6,8}$ = 2.4 Hz, H-6), 6.75 (d, 1H, H-8), 6.10 (brq, 1H, $J_{3,H}$ = 0.9 Hz, H-3), 5.95 (brs, 1H, NH), 4.14 (t, 2H, $J_{H,H}$ = 5.7 Hz, CH_2O), 3.72 (brt, 2H, $J_{H,H}$ = 6.4 Hz, CH_2N), 2.35 (d, 3H, CH_3), 2.22 (m, 2H, CH_2) ppm; ^{13}C-NMR (75.5 MHz, $CDCl_3$) δ 162.2 (C-7), 161.8 (C-2), 161.4 (C-2’’), 155.2 (C-8a), 152.7 (C-4), 148.6 (C-7’’a), 142.9 (C-3’’a), 125.6 (C-5), 124.0 (Ar-C), 121.0 (Ar-C), 116.3 (Ar-C), 113.8 (C-4a), 112.5 (C-6), 112.1 (C-3), 108.8 (Ar-C), 101.5 (C-8), 66.2 (CH_2O), 40.4 (CH_2N), 29.0 ($\mathit{C}H_2CH_2O$), 18.7 (CH_3) ppm; HRESI-MS *m/z* calcd. for $C_{20}H_{19}N_2O_4$ ($[M+H]^+$): 351.1339, found: 351.1336.

S2. 7-[{5'-(Benzo[*d*]oxazol-2''-ylamino)pentyl}oxy]-4-methyl-2*H*-chromen-2-one (8b). Method B. Isothiocyanate **7a** (126.6 mg, 0.42 mmol, 1.0 equiv.), 2-aminophenol (45.5 mg, 0.42 mmol, 1.0 equiv.), 30% H_2O_2 (85 μL, 0.83 mmol, 2.0 equiv.) and TBAI (2.6% mol, 4 mg) were used. Yield: 107 mg (67%, 2 steps), reddish foam. R_f 0.33 (1:1 Cyclohexane–EtOAc); ^{1}H-NMR (500 MHz, $CDCl_3$) δ 7.46 (d, 1H, $J_{5,6}$ = 8.8 Hz, H-5), 7.36 (m, 1H, Ar-H), 7.23 (m, 1H, Ar-H), 7.15 (td, 1H, $J_{H,H}$ = 7.7 Hz, $J_{H,H}$= 1.1 Hz, Ar-H), 7.02 (td, 1H, $J_{H,H}$ = 7.8 Hz, $J_{H,H}$= 1.2 Hz, Ar-H), 6.83 (dd, 1H, $J_{6,8}$ = 2.5 Hz, H-6), 6.79 (d, 1H, H-8), 6.13 (brq, 1H, $J_{3,H}$ = 1.2 Hz, H-3), 5.05 (s, 1H, NH), 4.02 (t, 2H, $J_{H,H}$ = 6.3 Hz, CH_2O), 3.53 (q, 2H, $J_{H,H}$ = $J_{H,NH}$ = 6.8 Hz, CH_2N), 2.39 (d, 3H, CH_3), 1.88 (m, 2H, CH_2), 1.78 (m, 2H, CH_2), 1.62 (m, 2H, CH_2) ppm; ^{13}C-NMR (125.7 MHz, $CDCl_3$) δ 162.0 (x2) (C-2, C-7), 161.5 (C-2''), 155.4 (C-8a), 152.7 (C-4), 148.7 (C-7''a), 143.1 (C-3''a), 125.6 (C-5), 124.0 (Ar-C), 121.0 (Ar-C), 116.5 (Ar-C), 113.7 (C-4a), 112.8 (C-6), 112.0 (C-3), 108.8 (Ar-C), 101.5 (C-8), 68.3 (CH_2O), 43.2 (CH_2N), 29.6 ($\mathit{C}H_2CH_2O$), 28.8 ($\mathit{C}H_2CH_2N$), 23.4 (CH_2), 18.8 (CH_3) ppm; HRESI-MS *m/z* calcd. for $C_{22}H_{23}N_2O_4$ ($[M+H]^+$): 379.1652, found: 379.1646.

S3. 7-[{6'-(Benzo[*d*]oxazol-2''-ylamino)hexyl}oxy]-4-methyl-2*H*-chromen-2-one (8c). Method B. Isothiocyanate **7c** (106.3 mg, 0.34 mmol, 1.0 equiv.), 2-aminophenol (36.5 mg, 0.34 mmol, 1.0 equiv.), 30% H_2O_2 (68 µL, 0.67 mmol, 2.0 equiv.) and TBAI (2.6% mol, 3 mg) were added. Yield: 65.7 mg (49%, 2 steps), reddish foam. R_f 0.40 (1:1 Cyclohexane–EtOAc); ^{1}H-NMR (500 MHz, $CDCl_3$) δ 7.47 (d, 1H, $J_{5,6}$ = 8.7 Hz, H-5), 7.36 (brd, 1H, $J_{H,H}$ = 6.1 Hz, Ar-H), 7.26 (m, 1H, Ar-H), 7.17 (t, 1H, $J_{H,H}$ = 7.6 Hz, Ar-H), 7.03 (t, 1H, $J_{H,H}$ = 7.7 Hz, Ar-H), 6.83 (dd, $J_{6,8}$ = 2.4 Hz, 1H, H-6), 6.79 (d, 1H, H-8), 6.13 (brq, 1H, $J_{3,H}$ = 1.0 Hz, H-3), 5.27 (brs, 1H, NH), 4.01 (t, 2H, $J_{H,H}$ = 6.3 Hz, CH_2O), 3.52 (brt, 2H, $J_{H,H}$ = 6.6 Hz, CH_2N), 2.39 (d, 3H, CH_3), 1.83 (quint, 2H, $J_{H,H}$ = 6.6 Hz, CH_2), 1.74 (quint, 2H, $J_{H,H}$ = 71 Hz, CH_2), 1.52 (m, 4H, 2CH_2) ppm; ^{13}C-NMR (125.7 MHz, $CDCl_3$) δ 162.3 (x2) (C-2, C-7), 161.5 (C-2''), 155.5 (C-8a), 152.7 (C-4), 125.6 (Ar-C), 124.2 (Ar-C), 121.2 (Ar-C), 116.4 (Ar-C), 113.6 (C-4a), 112.8 (C-6), 112.0 (C-3), 109.0 (Ar-C), 101.5 (C-8), 68.5 (CH_2O), 43.3 (CH_2N), 29.8 ($\mathit{C}H_2CH_2O$), 29.0 ($\mathit{C}H_2CH_2N$), 26.6 (CH_2), 25.8 (CH_2), 18.8 (CH_3) ppm; HRESI-MS *m/z* calcd. for $C_{23}H_{25}N_2O_4$ ($[M+H]^+$): 393.1809, found: 393.1804.

S4. 7-[{6’-(Benzo[*d*]oxazol-2-ylamino)hexyl}oxy]-3,4-dimethyl-2*H*-chromen-2-one (8d). Method A. Amino derivative **6d** (168.7 mg, 0.58 mmol, 1 equiv.), TCDI (136.1 mg, 0.76 mmol, 1.3 equiv.), DMAP (70.8 mg, 0.58 mmol, 1.0 equiv.), 2-aminophenol (63.2 mg, 0.51 mmol, 1.0 equiv.), 30% H_2O_2 (118 µL, 1.16 mmol, 2.0 equiv.) and TBAI (1.4% mol, 3 mg) were added. Yield: 80.5 mg (34%, 3 steps), reddish foam. R_f = 0.40 (1:1 Cyclohexane–EtOAc); ^{1}H-NMR (500 MHz, $CDCl_3$) δ= 7.46 (d, 1H, $J_{5,6}$ = 8.8 Hz, H-5), 7.35 (brd, 1H, $J_{H,H}$ = 7.6 Hz, Ar-H), 7.25 (brd, 1H, $J_{H,H}$ = 8.2 Hz, Ar-H), 7.16 (t, 1H, $J_{H,H}$ = 7.5 Hz, Ar-H), 7.02 (t, 1H, $J_{H,H}$ = 7.8 Hz, Ar-H), 6.83 (dd, 1H, $J_{6,5}$ = 8.8 H, $J_{6,8}$ = 2.5 Hz, H-6), 6.77 (d, 1H, H-8), 5.23 (brs, 1H, H-N<u>H</u>), 4.00 (t, 2H, $J_{H,H}$ = 6.4 Hz, CH_2O), 3.51 (brt, 2H, $J_{H,H}$ = 6.6 Hz, CH_2N), 2.36 (s, 3H, CH_3), 2.18 (s, 3H, CH_3), 1.82 (m, 2H, CH_2), 1.73 (m, 2H, CH_2), 1.53 (m, 4H, 2CH_2) ppm; ^{13}C-NMR (125.7 MHz, $CDCl_3$) δ 162.6 (C-7, C-2), 161.1 (C-<u>C</u>=N), 153.7 (C-9), 146.5 (C-4), 125.3 (C-Ar-O, C-Ar-N, C-5), 124.1 (C-Ar), 121.1 (C-Ar), 119.0 (C-10), 116.4 (C-Ar), 114.2 (C-3), 112.6 (C-6), 108.9 (C-Ar), 101.2 (C-8), 68.4 (CH_2O), 43.2 (CH_2N), 29.8 (*C*H_2CH_2O), 29.1 (*C*H_2CH_2N), 26.6 (CH_2), 25.8 (CH_2), 15.2 (CH_3), 13.3 (CH_3) ppm; HRESI-MS *m/z* calcd. for $C_{24}H_{27}N_2O_4$ (**[**M+H]$^+$): 407.1965, found: 407.1958.

S5. 7-[{6'-(Benzo[*d*]oxazol-2-ylamino)hexyl}oxy]-3-chloro-4-methyl-2*H*-chromen-2-one (8e).

Method B. Isothiocyanate **7e** (149.4 mg, 0.43 mmol, 1.0 equiv.), 2-aminophenol (46.4 mg, 0.43 mmol, 1.0 equiv.), 30% H_2O_2 (88 µl, 0.85 mmol, 2.0 equiv.) and TBAI (1.9% mol, 3 mg) were added. Yield 45.1 mg (25%, 2 steps), reddish foam. R_f = 0.41 (1:1 Cyclohexane–EtOAc); ^{1}H-NMR (500 MHz, $CDCl_3$) δ 7.49 (d, 1H, $J_{5,6}$ = 8.9 Hz, H-5), 7.36 (brd, 1H, $J_{H,H}$ = 7.9 Hz, Ar-H), 7.23 (brd, 1H, $J_{H,H}$ = 7.7 Hz, Ar-H), 7.16 (td, 1H, $J_{H,H}$ = 7.8 Hz, $J_{H,H}$ = 1.1 Hz, Ar-H), 7.03 (td, 1H, $J_{H,H}$ = 7.8 Hz, $J_{H,H}$ = 1.2 Hz, Ar-H), 6.87 (dd, 1H, $J_{5,6}$ = 8.9 Hz, $J_{6,8}$ = 2.5 Hz, H-6), 6.80 (d, 1H, $J_{8,6}$ = 2.5 Hz, H-8), 4.97 (brs, 1H, NH), 4.02 (t, 2H, $J_{H,H}$ = 6.3 Hz, CH_2O), 3.51 (q, 2H, $J_{H,H}$ = $J_{H,NH}$ = 6.8 Hz, CH_2N), 2.54 (s, 3H, CH_3), 1.84 (m, 2H, CH_2), 1.74 (m, 2H, CH_2), 1.54 (m, 4H, $2CH_2$) ppm; ^{13}C-NMR (125.7 MHz, $CDCl_3$) δ 162.2 (C-7, C-2), 157.6 (C=N), 153.3 (C-9), 148.6 (C-4), 148.1 (Ar-C), 143.1 (Ar-C), 126.0 (C-5), 124.0 (Ar-C), 121.0 (Ar-C), 117.8 (Ar-C), 116.5 (C-3), 113.5 (C-6), 113.3 (C-10), 108.8 (Ar-C), 101.4 (C-8), 68.6 (CH_2O), 43.2 (CH_2N), 29.8 ($\mathit{C}H_2CH_2O$), 29.0 ($\mathit{C}H_2CH_2N$), 26.6 (CH_2), 25.8 (CH_2), 16.3 (CH_3) ppm; HRESI-MS *m/z* calcd. for $C_{23}H_{24}ClN_2O_4$ ($[M+H]^+$): 427.1419, found: 427.1412.

S6. 7-[{6'-(Benzo[*d*]oxazol-2-ylamino)hexyl}oxy]-3-methyl-4-phenyl-2*H*-chromen-2-one (8f).

Method B. Isothiocyanate **7f** (147.5 mg, 0.39 mmol, 1.0 equiv.), 2-aminophenol (42.4 mg, 0.39 mmol, 1.0 equiv.), 30% H_2O_2 (80 µL, 0.78 mmol, 2.0 equiv.) and TBAI (2% mol, 3 mg) were added. Yield: 52.8 mg (30%, 2 steps), reddish foam. R_f = 0.41 (1:1 Cyclohexane–EtOAc); ^{1}H-NMR (500 MHz, $CDCl_3$) δ 7.53–7.49 (m, 3H, Ar-H Ph), 7.45–7.42 (m, 2H, Ar-H, Ph), 7.36 (d, 2H, $J_{5,6}$ = 8.9 Hz, H-5, Ar-H), 7.23 (brdd, 1H, $J_{H,H}$ = 8.2 Hz, $J_{H,H}$ = 0.9 Hz, Ar-H), 7.16 (td, 1H, $J_{H,H}$ = 7.8 Hz, $J_{H,H}$ = 1.0 Hz, Ar-H), 7.03 (td, 1H, $J_{H,H}$ = 8.0 Hz, $J_{H,H}$ = 1.0 Hz, Ar-H), 6.87 (d, 1H, $J_{8,6}$ = 2.3 Hz, H-8), 6.77 (dd, $J_{6,5}$ = 8.9 H, $J_{6,8}$ = 2.3 Hz, 1H, H-6), 6.21 (s, 1H, H-3), 5.00 (brs, 1H, NH), 4.03 (t, 2H, $J_{H,H}$ = 6.4 Hz, CH_2O), 3.51 (q, 2H, $J_{H,H}$ = $J_{H,NH}$ = 6.9 Hz, CH_2N), 1.84 (m, 2H, CH_2), 1.74 (m, 2H, CH_2), 1.53 (m, 4H, 2CH_2) ppm; ^{13}C-NMR (125.7 MHz, $CDCl_3$) δ 162.4 (C-7), 162.1 (C-2), 161.4 (C=N), 156.2 (C-9), 156.0 (C-4), 148.6 (Ar-C), 143.1 (Ar-C), 135.8 (Ar-C, Ph), 129.7 (Ar-C, Ph), 129.0 (Ar-C, Ph), 128.5 (Ar-C, Ph), 128.1 (C-5), 124.0 (Ar-C), 121.0 (Ar-C), 116.5 (Ar-C), 112.8 (C-6), 112.6 (C-10), 111.9 (C-3), 108.8 (Ar-C), 101.7 (C-8), 68.5 (CH_2O), 43.2 (CH_2N), 29.8 ($\mathit{C}H_2CH_2O$), 29.0 ($\mathit{C}H_2CH_2N$), 26.6 (CH_2), 25.8 (CH_2) ppm; HRESI-MS *m/z* calcd. for $C_{28}H_{27}N_2O_4$ **(**[M+H]$^+$): 455.1965, found: 455.1959.

S.7 7-[{5'-((5''-Methylbenzo[*d*]oxazol-2-yl)amino)pentyl}oxy]-4-methyl-2*H*-chromen-2-one (8g).

Method B. Isothiocyanate **7a** (104 mg, 0.34 mmol, 1.0 equiv.), 2-amino-4-methylphenol (37.1 mg, 0.39 mmol, 1.0 equiv.), 30% H_2O_2 (70 µL, 0.69 mmol, 2.0 equiv.) and TBAI (3% mol, 4 mg) were added. Yield: 57 mg (43%, 2 steps), reddish foam. R_f = 0.36 (1:1 Cyclohexane–EtOAc); ^{1}H-NMR (500 MHz, $CDCl_3$) δ 7.46 (d, 1H, $J_{5,6}$ = 8.8 Hz, H-5), 7.14 (brs, 1H, Ar-H), 7.09 (d, 1H, $J_{H,H}$ = 7.8 Hz, Ar-H), 6.82 (dd, 2H, $J_{5,6}$ = 8.8 Hz, $J_{6,8}$ = 2.5 Hz, H-6, Ar-H), 6.77 (d, 1H, $J_{6,8}$ = 2.5 Hz, H-8), 6.12 (brs, 1H, H-3), 5.27 (s, 1H, NH), 4.01 (t, 2H, $J_{H,H}$ = 6.3 Hz, CH_2O), 3.51 (brt, 2H, $J_{H,H}$ = 6.7 Hz, CH_2N), 2.38 (brs, 6H, $2CH_3$), 1.87 (quint, 2H, $J_{H,H}$ = 6.7 Hz, CH_2), 1.77 (quint, 2H, $J_{H,H}$ = 7.2 Hz, CH_2), 1.60 (brquint, 2H, $J_{H,H}$= 6.7 Hz, CH_2) ppm; ^{13}C-NMR (125.7 MHz, $CDCl_3$) δ 162.4 (C-7), 162.2 (C-2), 161.5 (C=N), 155.4 (C-9), 152.7 (C-4), 146.7 (Ar-C), 143.2 (Ar-C), 133.7 (Ar-C), 125.6 (C-5), 121.5 (Ar-C), 116.8 (C-Ar), 113.6 (C-10), 112.7 (C-6), 112.0 (C-3), 108.2 (Ar-C), 101.5 (C-8), 68.4 (CH_2O), 43.1 (CH_2N), 29.6 ($\mathit{C}H_2CH_2O$), 28.8 ($\mathit{C}H_2CH_2N$), 23.4 (CH_2), 21.6 (CH_3) 18.8 (CH_3) ppm; HRESI-MS *m/z* calcd. for $C_{23}H_{25}N_2O_4$ ([M+H]$^+$): 393.1809, found: 393.1805.

S8. 7-[{5'-((5''-Bromobenzo[*d*]oxazol-2-yl)amino)pentyl}oxy]-4-methyl-2*H*-chromen-2-one (8h).

Method B. Isothiocyanate **7a** (104 mg, 0.34 mmol, 1.0 equiv.), 2-amino-4-bromophenol (64.4 mg, 0.59 mmol, 1.7 equiv.), 30% H_2O_2 (70 µL, 0.69 mmol, 2.0 equiv.) and TBAI (3%, 4 mg) were added. Yield: 52.2 mg, (34%, 2 steps), reddish foam. R_f = 0.60 (1:1 Cyclohexane–EtOAc); ^{1}H-NMR (500 MHz, $CDCl_3$) δ 7.46 (d, 1H, $J_{5,6}$ = 8.8 Hz, H-5), 7.45 (d, 1H, $J_{4'',6''}$ = 1.9 Hz, H-4''), 7.12 (dd, 1H, $J_{6'',7''}$ = 8.4 Hz, H-6''), 7.08 (d, 1H, H-7''), 6.82 (dd, 1H, $J_{5,6}$ = 8.8 Hz, $J_{6,8}$ = 2.5 Hz, H-6), 6.77 (d, 1H, $J_{8,6}$ = 2.5 Hz, H-8), 6.12 (brq, 1H, $J_{3,H}$ = 1.2 Hz, H-3), 5.29 (brs, 1H, NH), 4.02 (t, 2H, $J_{H,H}$ = 6.3 Hz, CH_2O), 3.52 (brq, 2H, $J_{H,H}$ = $J_{H,NH}$ = 6.7 Hz, CH_2N), 2.39 (d, 3H, $J_{H,3'}$ = 1.2 Hz, CH_3), 1.88 (m, 2H, CH_2), 1.77 (m, 2H, CH_2), 1.61 (m, 2H, CH_2) ppm; ^{13}C-NMR (125.7 MHz, $CDCl_3$) δ 162.9 (C-7), 162.2 (C-2), 161.5 (C=N), 155.4 (C-9), 152.7 (C-4), 147.7 (Ar-C), 144.9 (Ar-C), 125.6 (C-5), 123.6 (Ar-C), 119.5 (Ar-C), 116.7 (Ar-C), 113.7 (C-10), 112.7 (C-6), 112.0 (C-3), 109.9 (Ar-C), 101.5 (C-8), 68.3 (CH_2O), 43.2 (CH_2N), 29.5 (*C*H_2CH_2O), 28.7 (*C*H_2CH_2N), 23.4 (CH_2), 18.8 (CH_3) ppm; HRESI-MS *m/z* calcd. for $C_{22}H_{22}{}^{79}BrN_2O_4$ ($[M+H]^+$): 457.0757, found: 457.0753.

S9. 7-[{5’-((5’’-Phenylbenzo[*d*]oxazol-2-yl)amino)pentyl}oxy]-4-methyl-2*H*-chromen-2-one (8i).

Method B. Isothiocyanate **7a** (97 mg, 0.32 mmol, 1.0 equiv.), 2-amino-4-phenylphenol (59.2 mg, 0.54 mmol, 1.7 equiv.), 30% H_2O_2 (65 µL, 0.64 mmol, 2.0 equiv.) and TBAI (3.4% mol, 4 mg) were added. Yield: 71.9 mg (49%, 2 steps), green foam. R_f = 0.40 (1:1 Cyclohexane–EtOAc); ^{1}H-NMR (500 MHz, $CDCl_3/CD_3OD$) δ 7.57 (m, 3H, Ar-H, Ph), 7.47 (d, 1H, $J_{5,6}$ = 8.8 Hz, H-5), 7.42 (m, 2H, Ar-H, Ph), 7.33 (tt, 1H, $J_{H,H}$ = 7.4 Hz, $J_{H,H}$ = 1.2 Hz, Ar-H), 7.27 (dd, 1H, $J_{H,H}$ = 8.4 Hz, $J_{H,H}$ = 0.5 Hz, Ar-H), 7.24 (dd, 1H, $J_{H,H}$ = 1.8 Hz, Ar-H), 6.84 (dd, 1H, $J_{5,6}$ = 8.8 Hz, $J_{6,8}$ = 2.4 Hz, H-6), 6.80 (d, 1H, $J_{6,8}$ = 2.54 Hz, H-8), 6.12 (brq, 1H, $J_{3,H}$ = 1.2 Hz, H-3), 4.03 (t, 2H, $J_{H,H}$ = 6.3 Hz, CH_2O), 3.53 (t, 2H, $J_{H,H}$ = 7.0 Hz, CH_2N), 2.38 (d, 3H, $J_{H,3}$ = 1.2 Hz, CH_3), 1.89 (quint, 2H, $J_{H,H}$ = 6.7 Hz, CH_2), 1.79 (quint, 2H, $J_{H,H}$ = 6.7 Hz, CH_2), 1.63 (m, 2H, CH_2) ppm; ^{13}C-NMR (125.7 MHz, $CDCl_3/CD_3OD$) δ 162.6 (C-7), 162.2 (C-2), 161.7 (C=N), 155.4 (C-9), 152.9 (C-4), 148.2 (Ar-C), 143.5 (Ar-C), 141.7 (Ar-C, Ph), 137.9 (Ar-C, Ph), 128.8 (Ar-C, Ph), 127.4 (Ar-C, Ph), 127.0 (Ar-C, Ph), 125.6 (C-5), 120.4 (Ar-C, Ph), 115.0 (Ar-C), 113.6 (C-10), 112.8 (C-6), 111.9 (C-3), 108.8 (Ar-C), 101.5 (C-8), 68.3 (CH_2O), 43.0 (CH_2N), 29.8 ($\mathit{C}H_2CH_2O$), 28.7 ($\mathit{C}H_2CH_2N$), 23.4 (CH_2), 18.8 (CH_3) ppm; HRESI-MS *m/z* calcd. for $C_{28}H_{27}N_2O_4$ **(**[M+H]$^+$): 455.1965, found: 455.1957.

S10. 7-[{5'-((5''-Sulfonamidobenzo[*d*]oxazol-2-yl)amino)pentyl}oxy]-4-methyl-2*H*-chromen-2-one (8j).

Method B. Isothiocyanate **7a** (100 mg, 0.33 mmol, 1.0 equiv.), 3-amino-4-hydroxybenzenesulfonamide (62.0 mg, 0.57 mmol, 1.7 equiv.), 30% H_2O_2 (67 μL, 0.66 mmol, 2.0 equiv.) and TBAI (3.3% mol, 4 mg) were added. Yield: 43.9 mg (30%, 2 steps), reddish foam. R_f = 0.40 (1:1 Cyclohexane–EtOAc). ^{1}H-NMR (500 MHz, $CD_3OD/CDCl_3$) δ 7.76 (d, 1H, $J_{4'',6''}$ = 1.9 Hz, H-4''), 7.59 (dd, 1H, $J_{6'',7''}$ = 8.3 Hz, H-6''), 7.57 (d, 1H, $J_{5,6}$ = 8.9 Hz, H-5), 7.30 (d, 1H, H-7''), 6.88 (dd, 1H, $J_{5,6}$ = 8.8 Hz, $J_{6,8}$ = 2.5 Hz, H-6), 6.81 (d, 1H, $J_{6,8}$ = 2.5 Hz, H-8), 6.12 (brq, 1H, $J_{3,H}$ = 1.2 Hz, H-3), 4.07 (t, 2H, $J_{H,H}$ = 6.3 Hz, CH_2O), 3.44 (t, 2H, $J_{H,H}$ = 7.0 Hz, CH_2N), 2.42 (d, 3H, $J_{H,3'}$ = 1.2 Hz, CH_3), 1.88 (quint, 2H, $J_{H,H}$ = 6.4 Hz, CH_2), 1.76 (quint, 2H, $J_{H,H}$ = 7.2 Hz, CH_2), 1.62 (m, 2H, CH_2) ppm; ^{13}C-NMR (125.7 MHz, $CD_3OD/CDCl_3$) δ 164.8 (C-7), 163.4 (C-2), 163.3 (C=N), 155.8 (C-9), 155.1 (C-4), 151.3 (Ar-C), 144.0 (Ar-C), 140.0 (Ar-C), 126.6 (C-5), 120.0 (Ar-C), 114.3 (Ar-C), 114.0 (C-10), 113.6 (C-6), 111.7 (C-3), 109.2 (C-Ar), 102.0 (C-8), 69.0 (CH_2O), 43.3 (CH_2N), 29.6 (CH_2CH_2O), 29.3 (CH_2CH_2N), 23.8 (CH_2), 18.8 (CH_3) ppm; HRESI-MS *m/z* calcd. for $C_{22}H_{24}N_3O_6S$ ($[M+H]^+$): 458.1380, found: 458.1371.

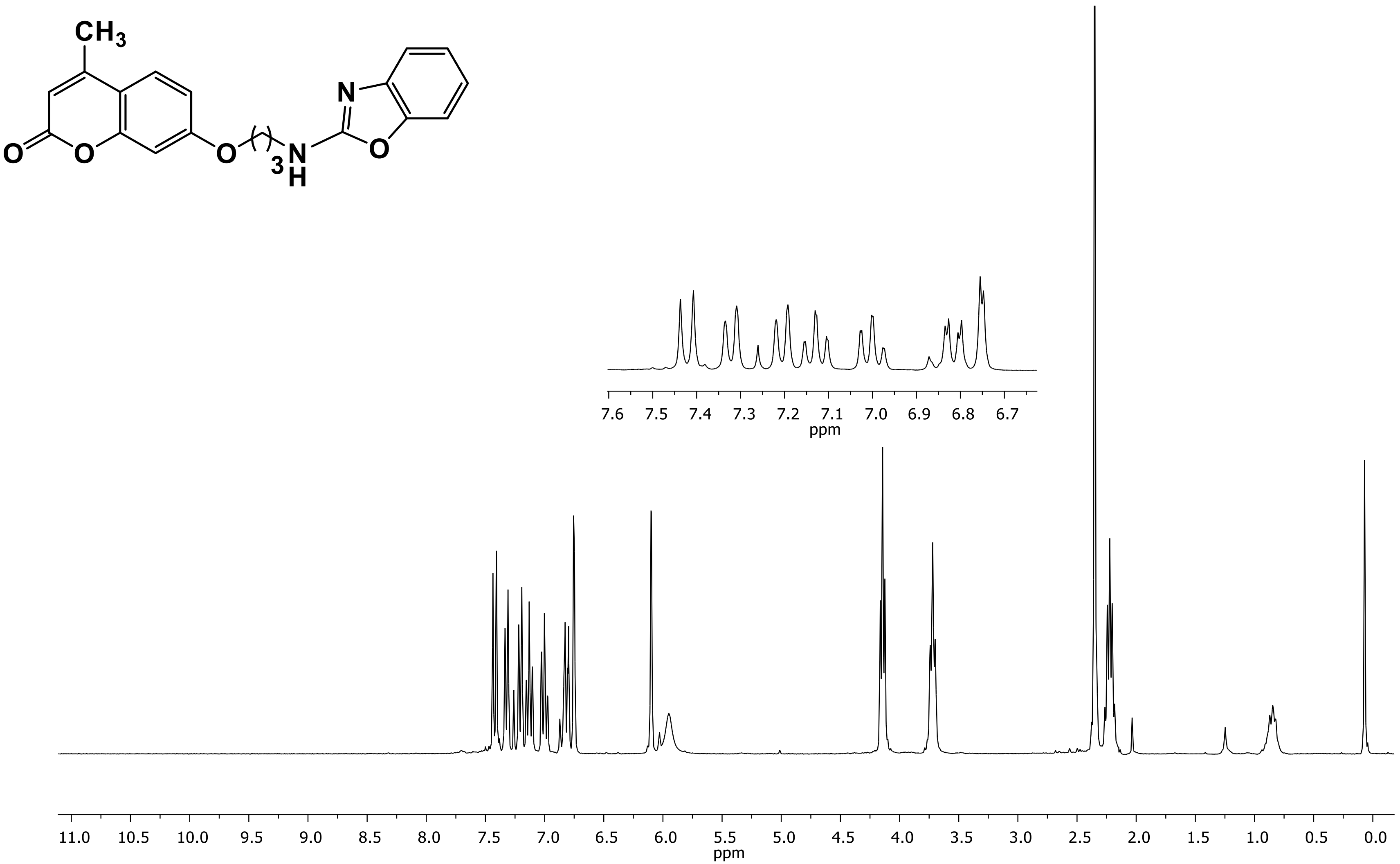

^{1}H-NMR (300 MHz, $CDCl_3$) of **8a**

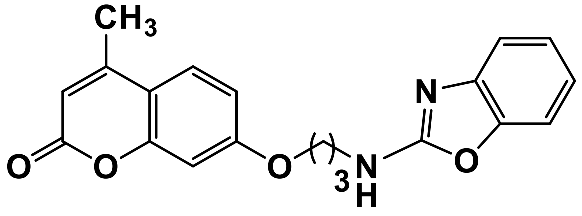

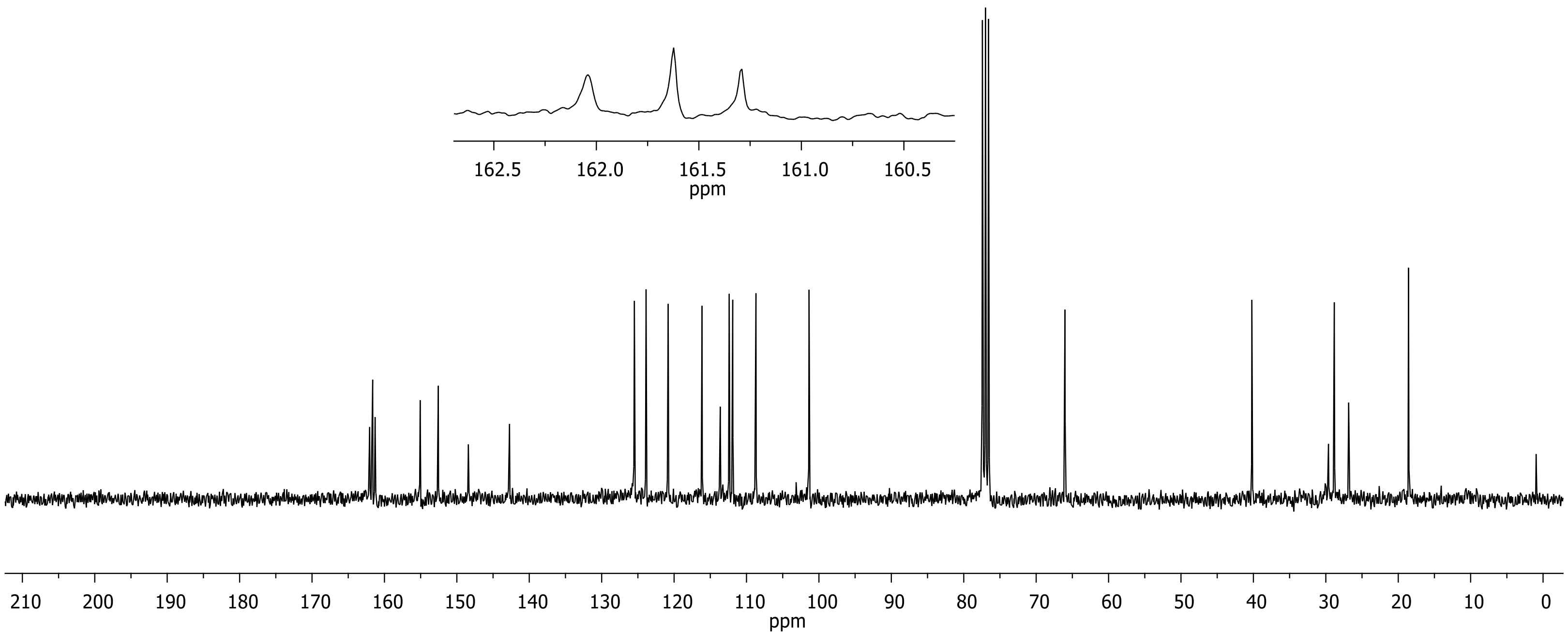

^{13}C-NMR (75.5 MHz, $CDCl_3$) of **8a**

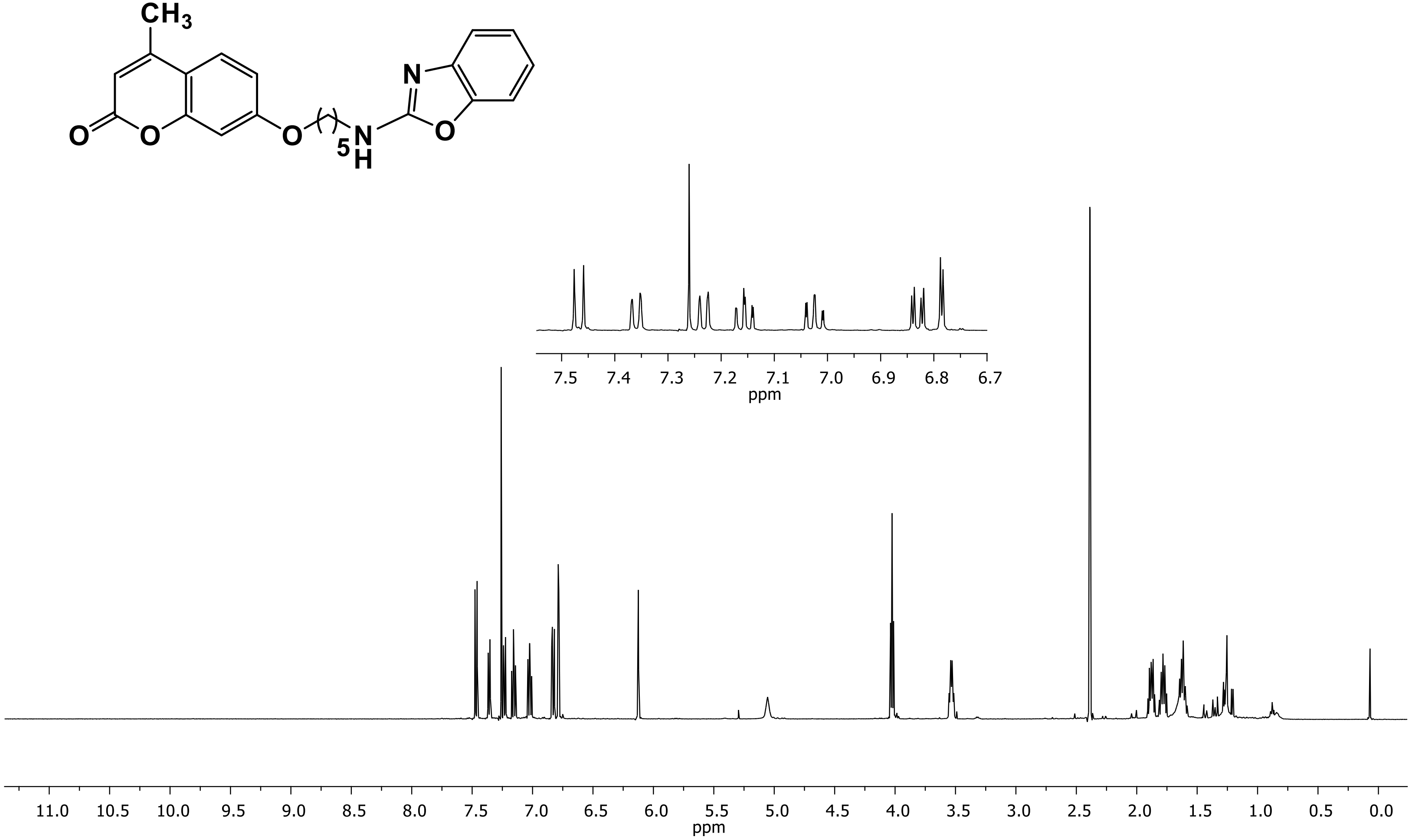

^{1}H-NMR (300 MHz, $CDCl_3$) of **8b**

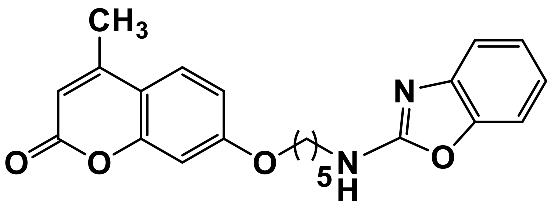

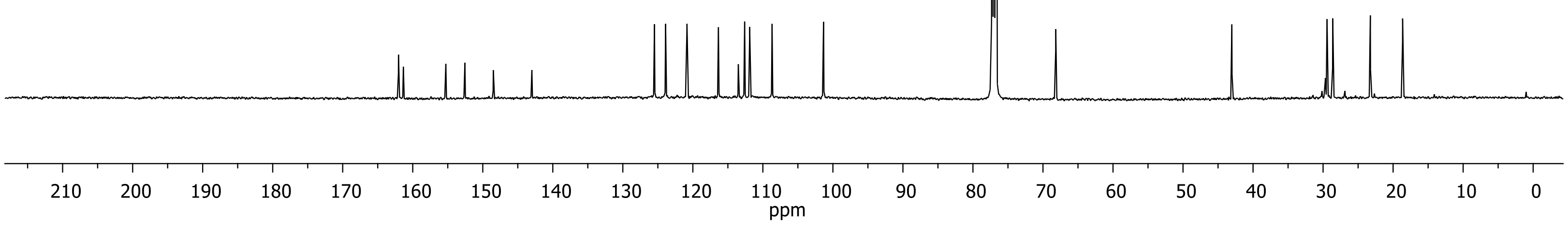

^{13}C-NMR (125.7 MHz, $CDCl_3$) of **8b**

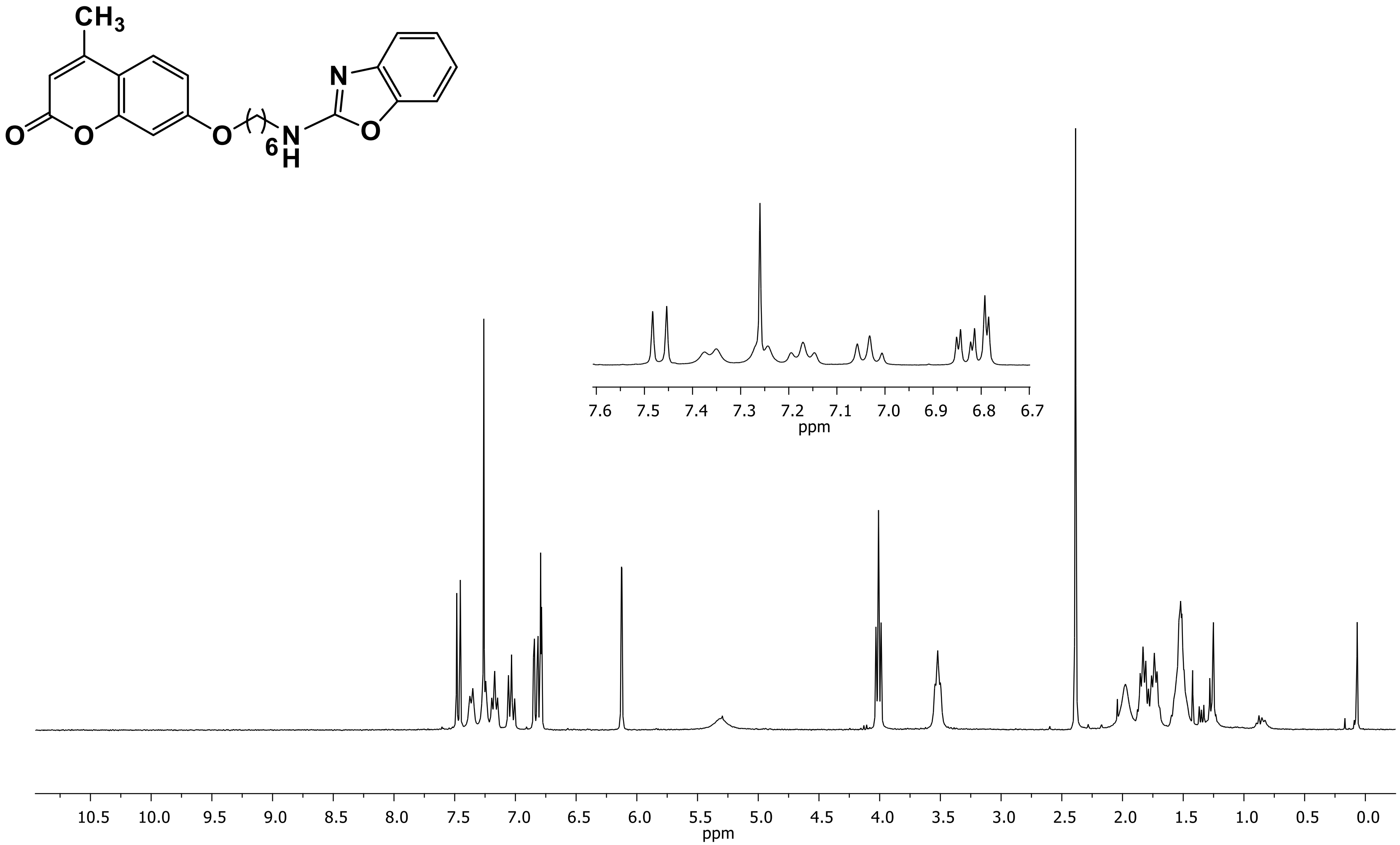

^{1}H-NMR (300 MHz, $CDCl_3$) of **8c**

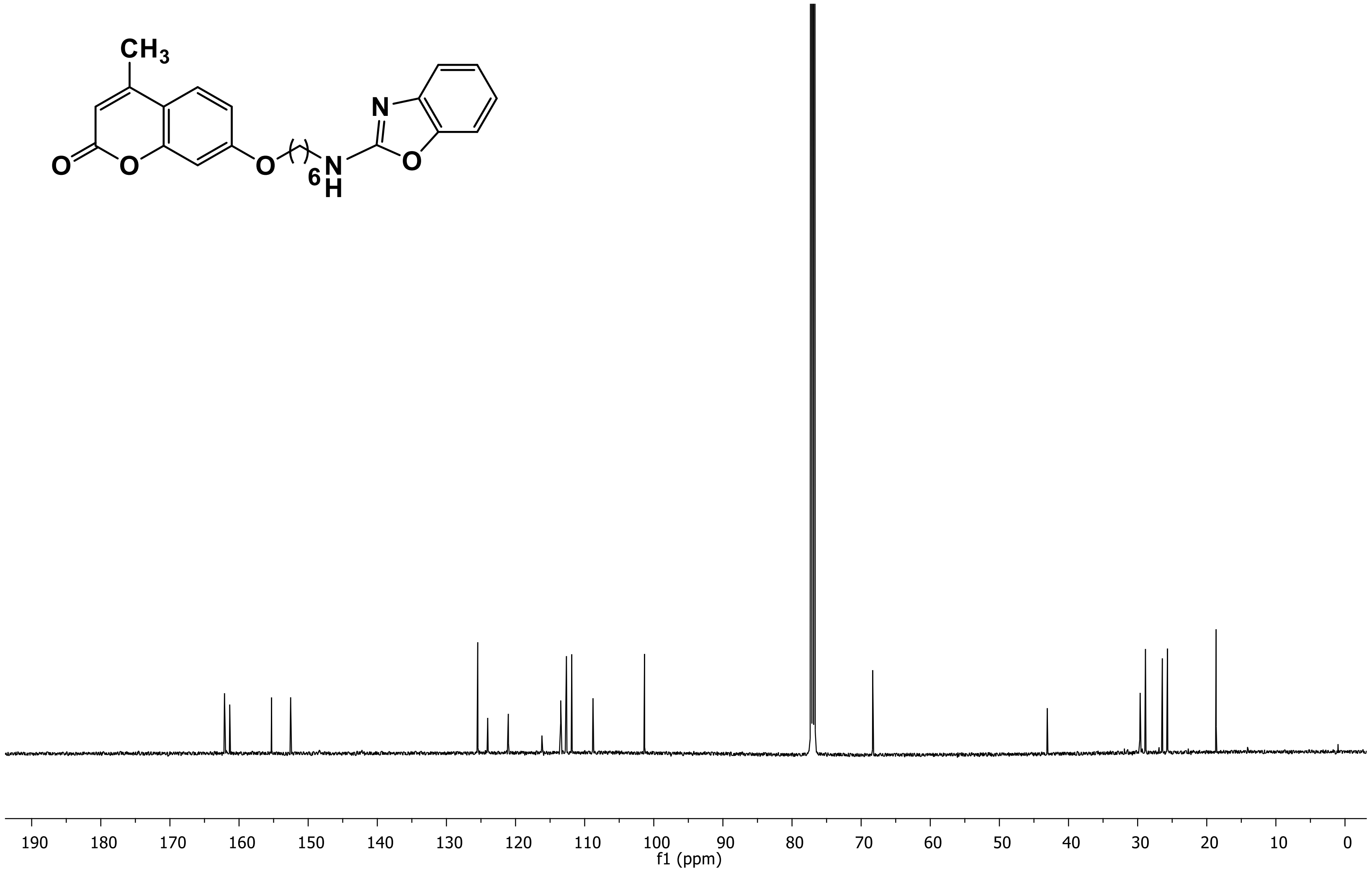

^{13}C-NMR (125.7 MHz, $CDCl_3$) of **8c**

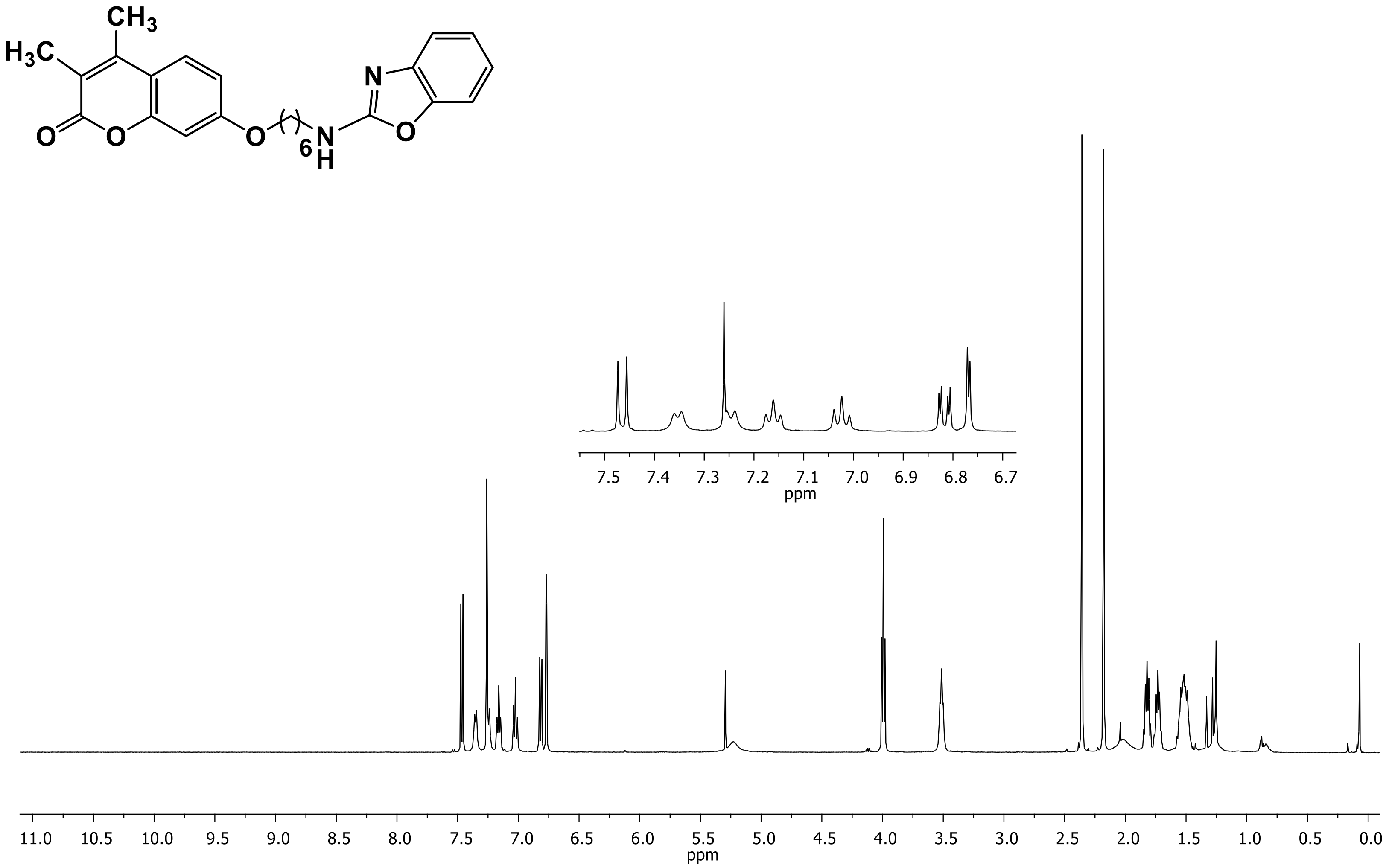

^{1}H-NMR (300 MHz, $CDCl_3$) of **8d**

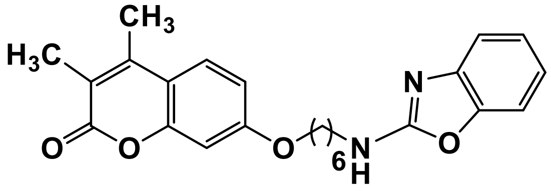

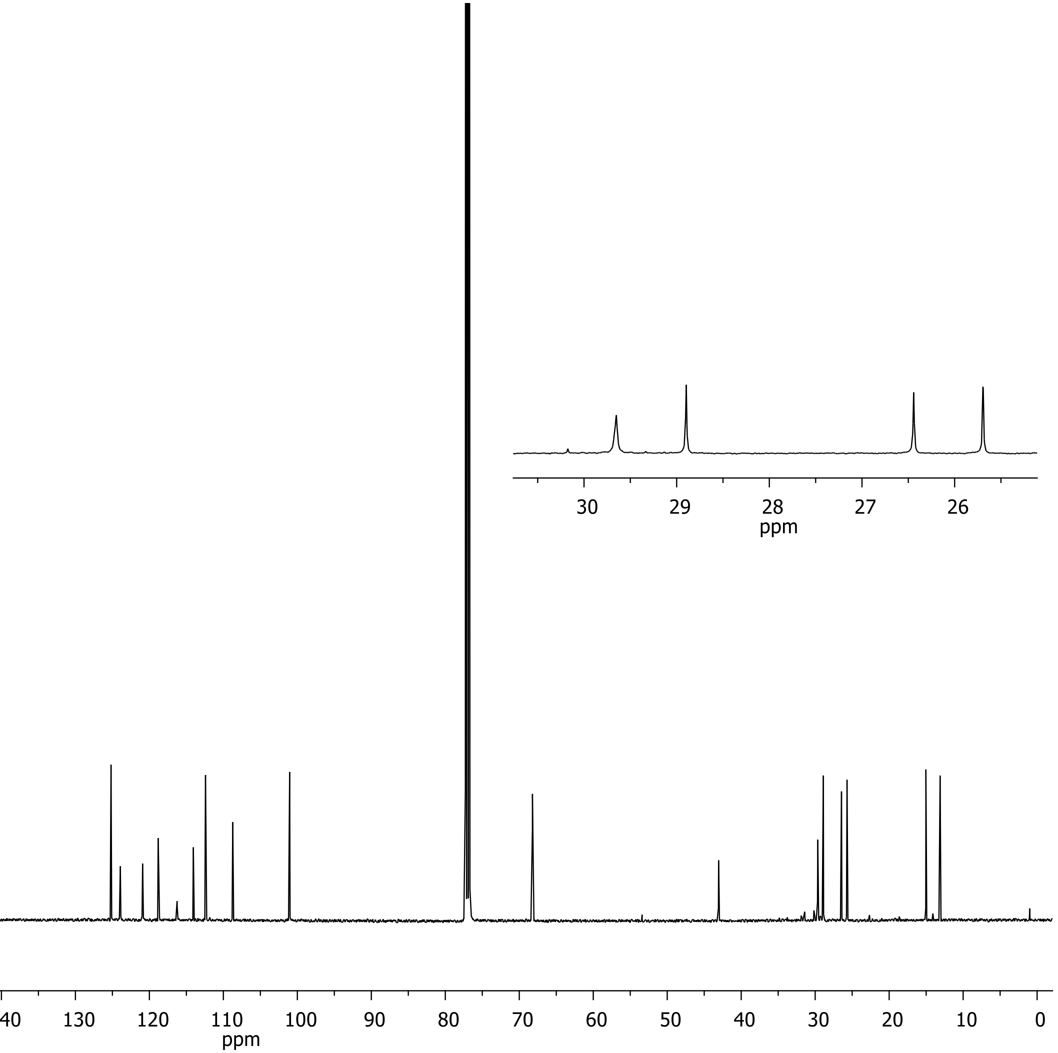

^{13}C-NMR (125.7 MHz, $CDCl_3$) of **8d**

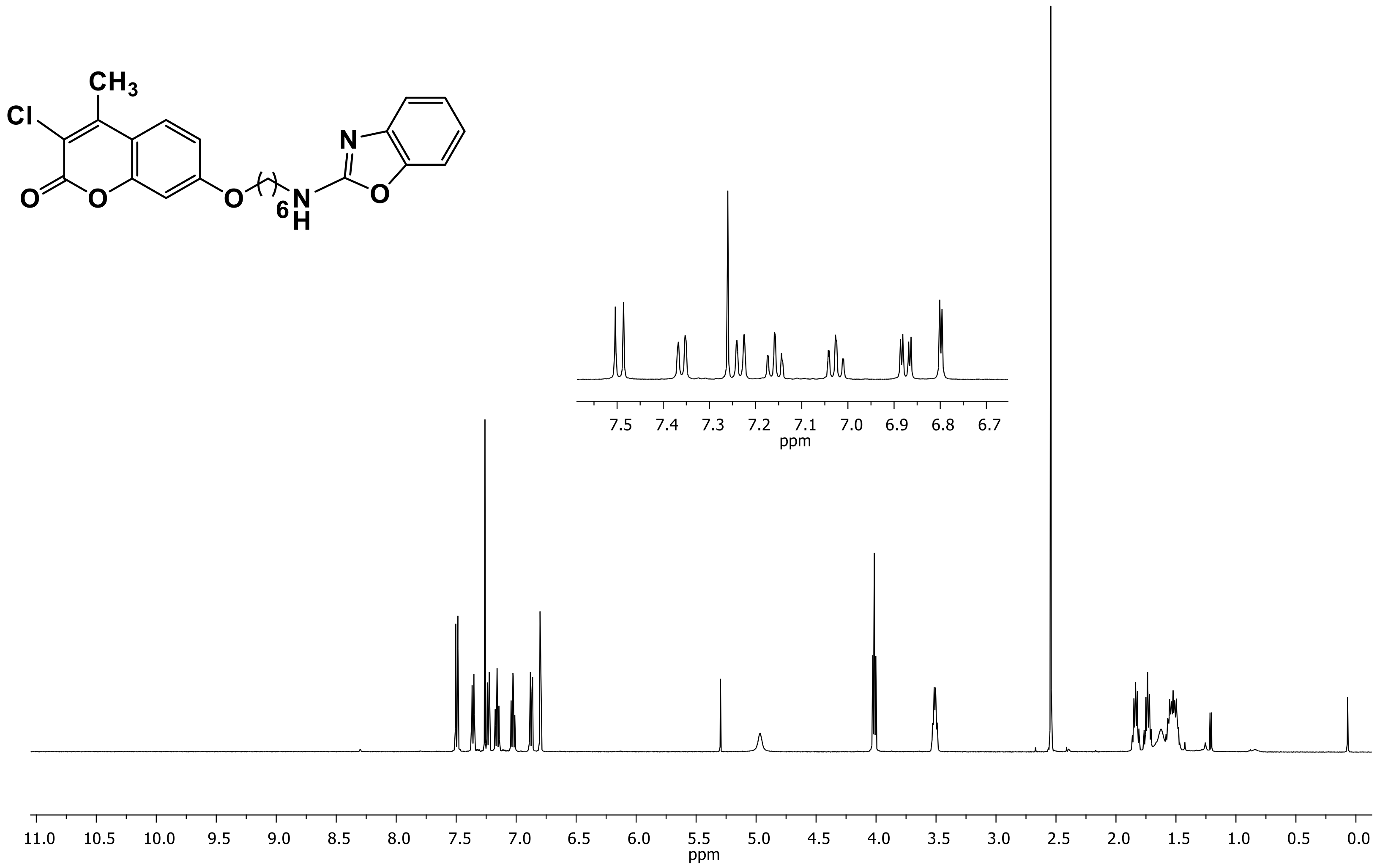

^{1}H-NMR (300 MHz, $CDCl_3$) of **8e**

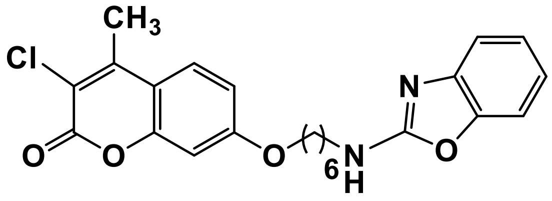

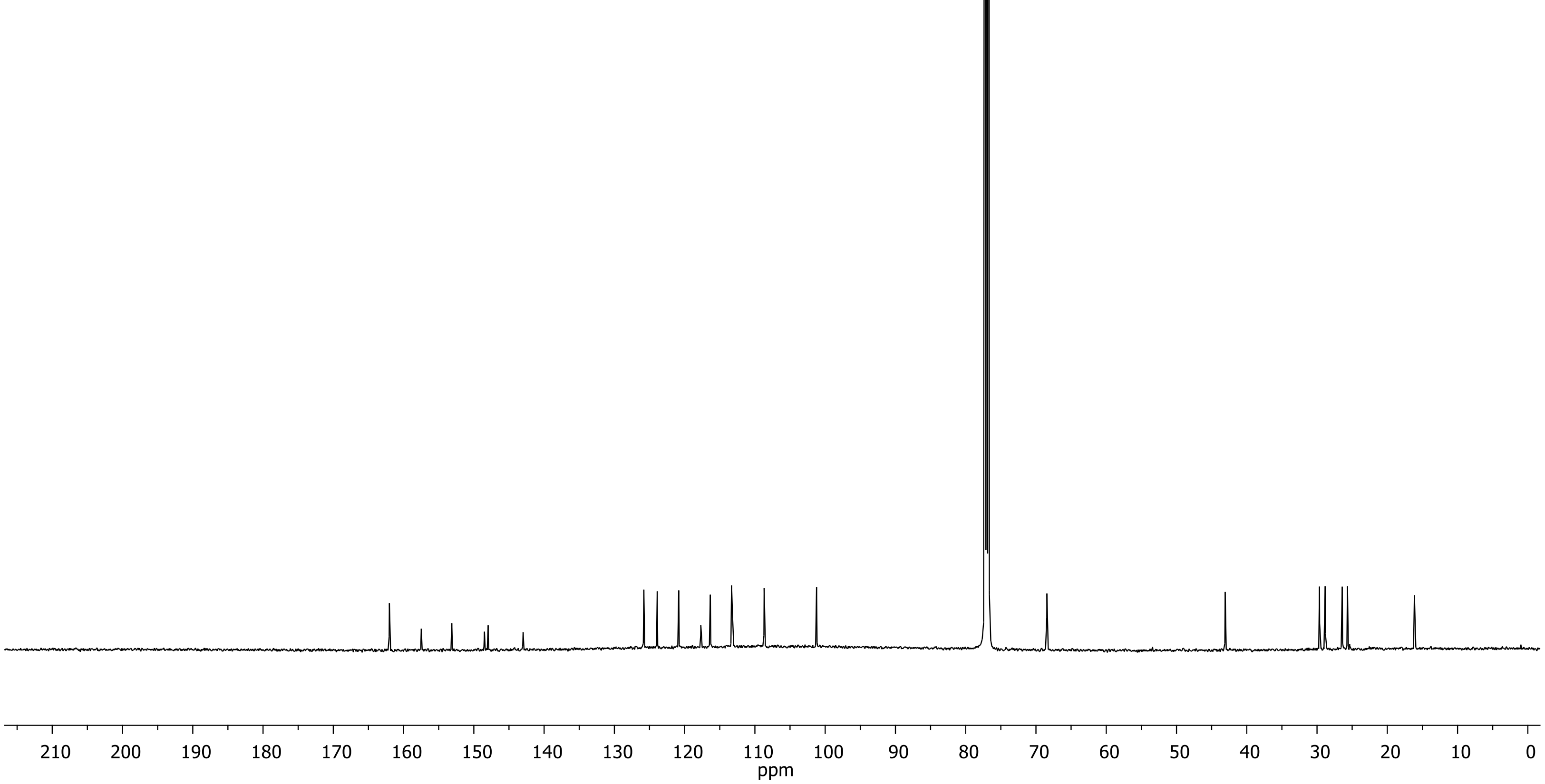

^{13}C-NMR (125.7 MHz, $CDCl_3$) of **8e**

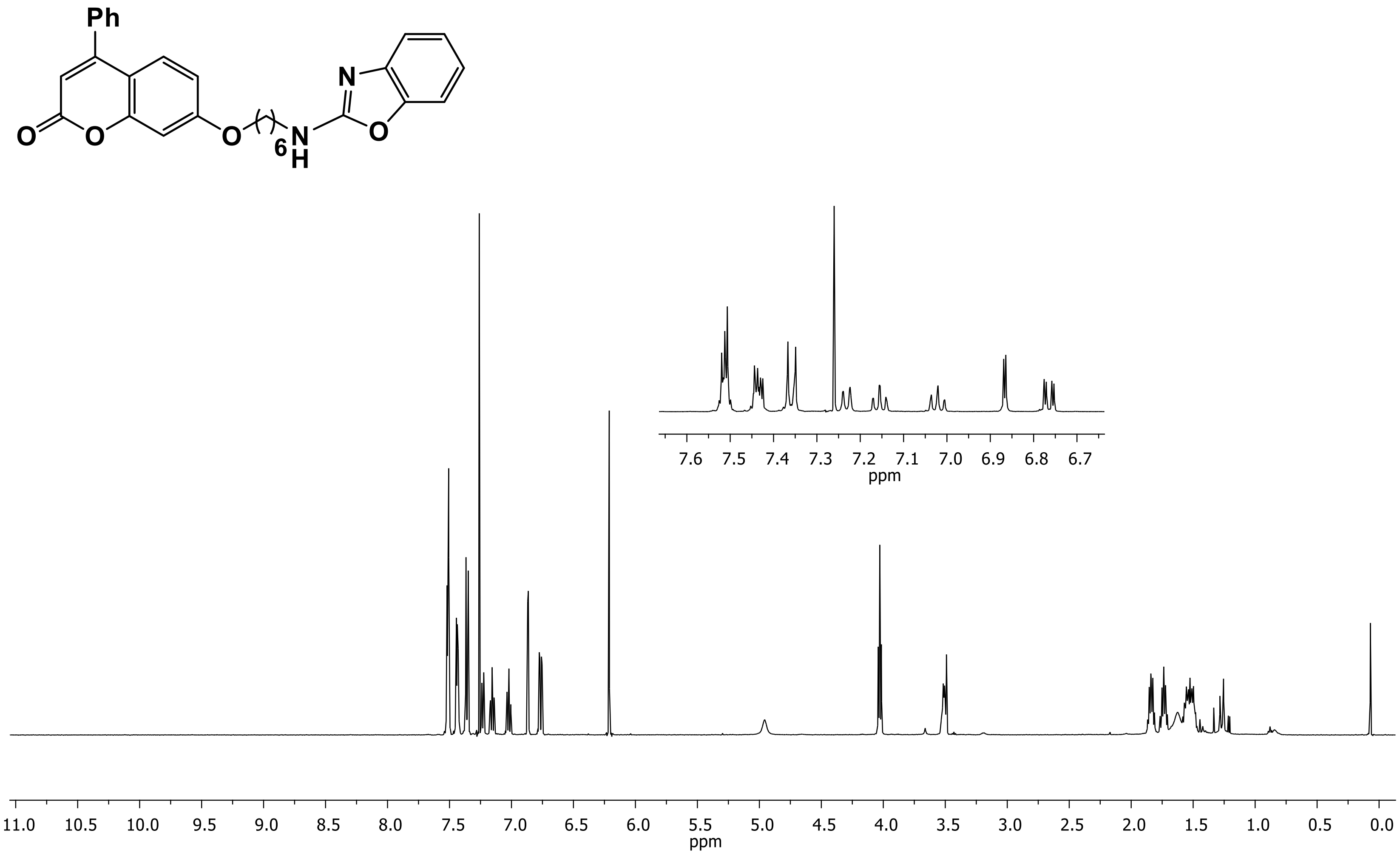

^{1}H-NMR (300 MHz, $CDCl_3$) of **8f**

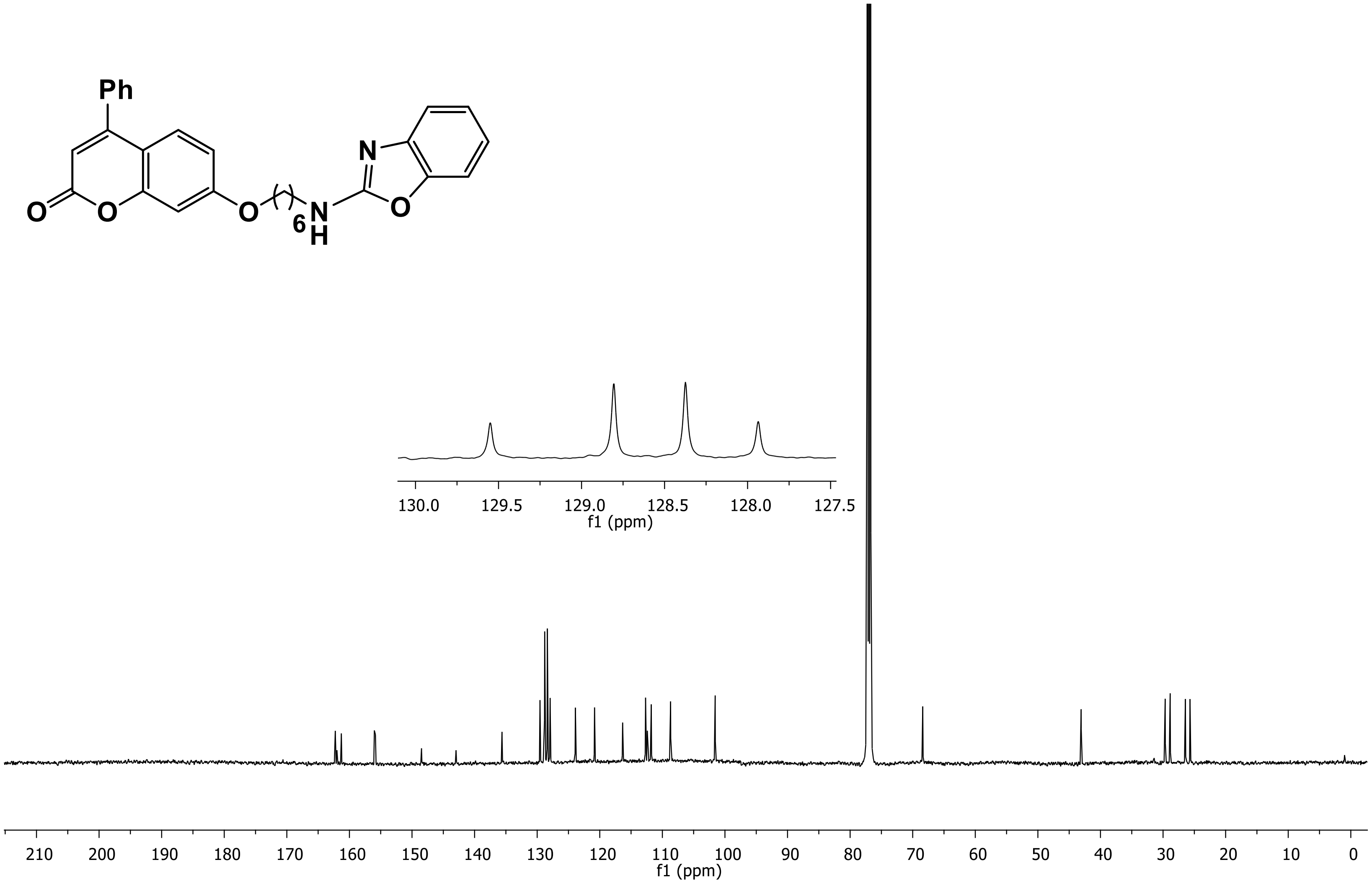

^{13}C-NMR (125.7 MHz, $CDCl_3$) of **8f**

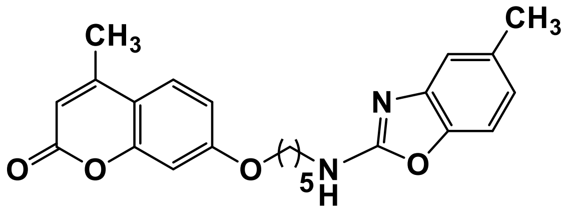

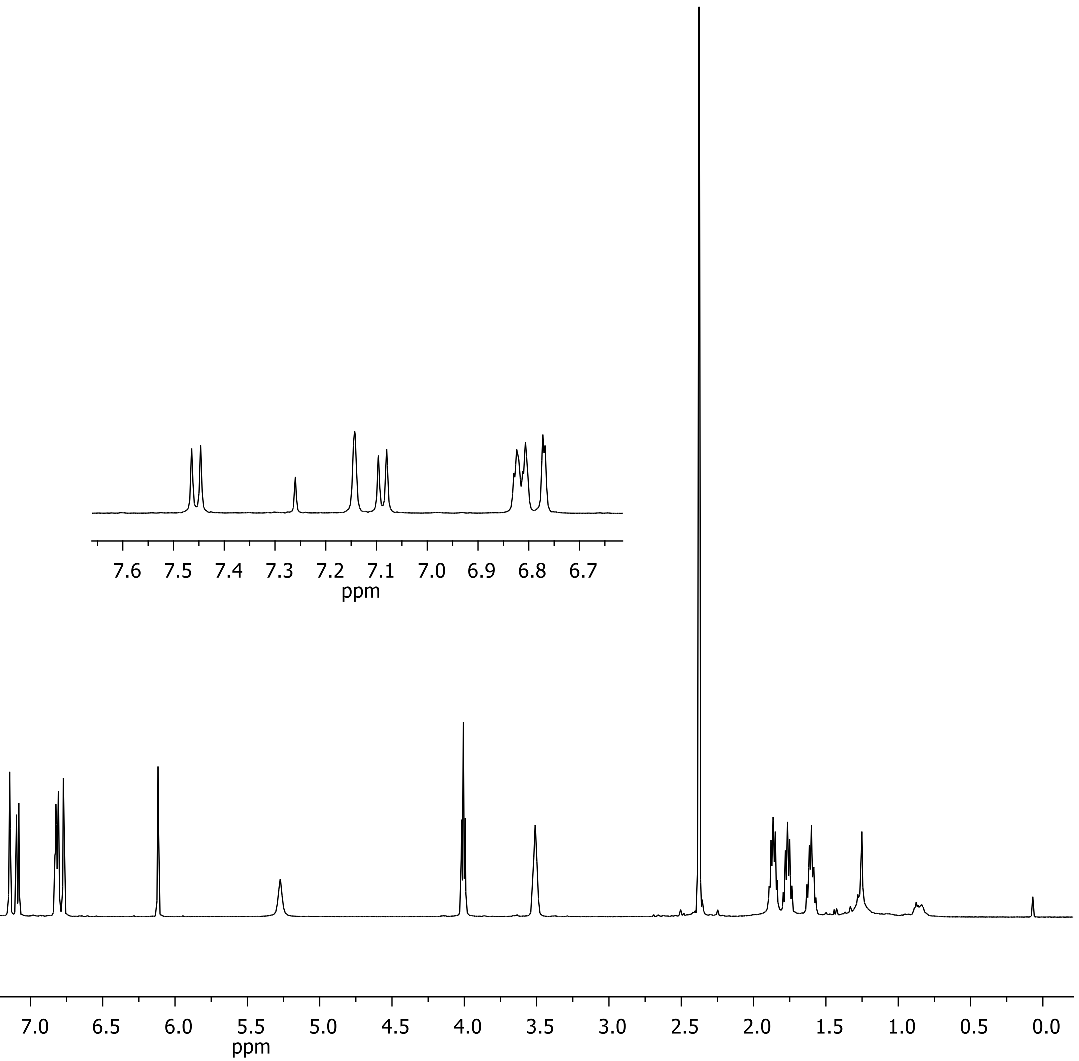

^{1}H-NMR (300 MHz, $CDCl_3$) of **8g**

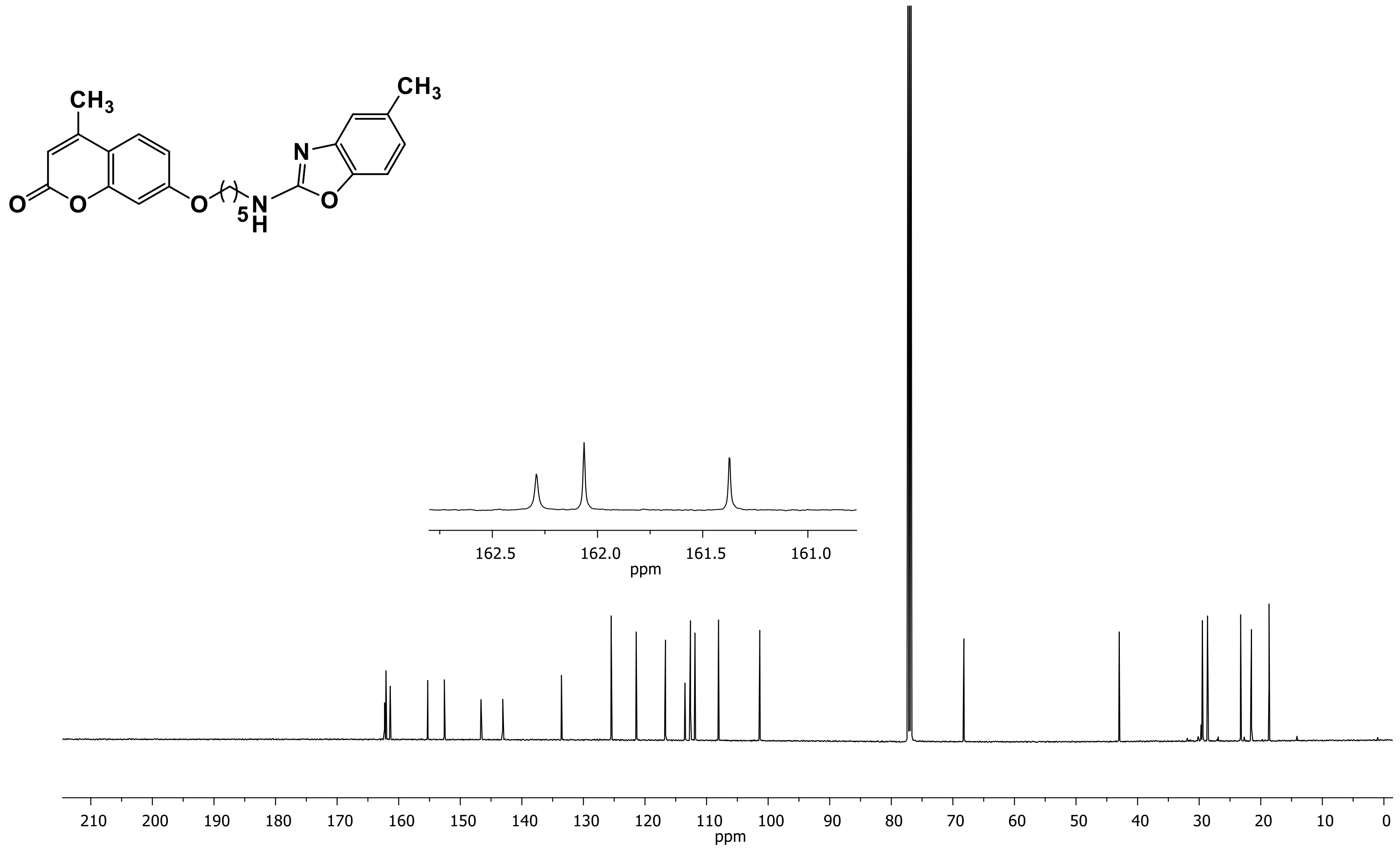

^{13}C-NMR (125.7 MHz, $CDCl_3$) of **8g**

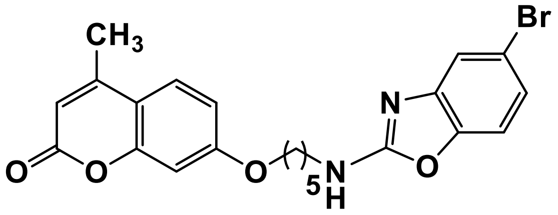

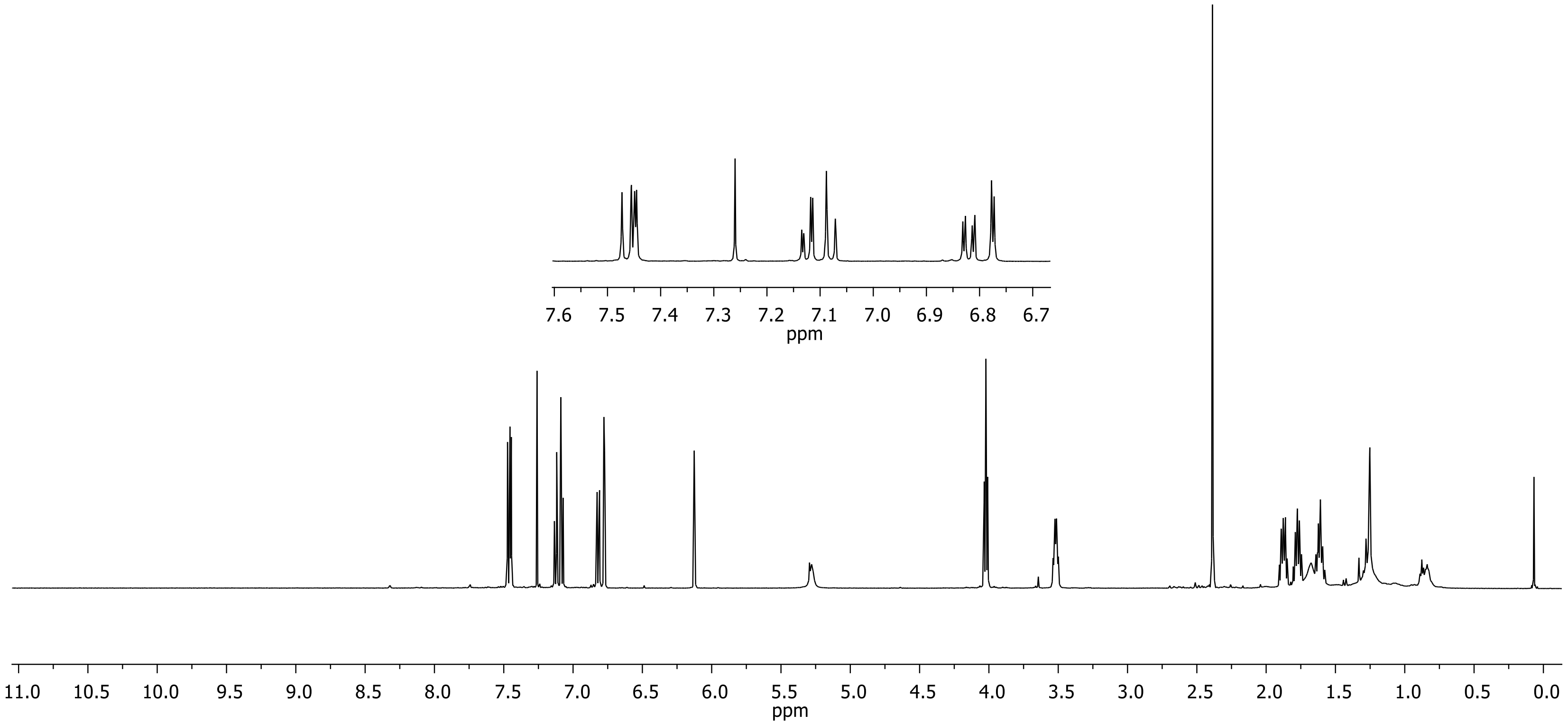

^{1}H-NMR (300 MHz, $CDCl_3$) of **8h**

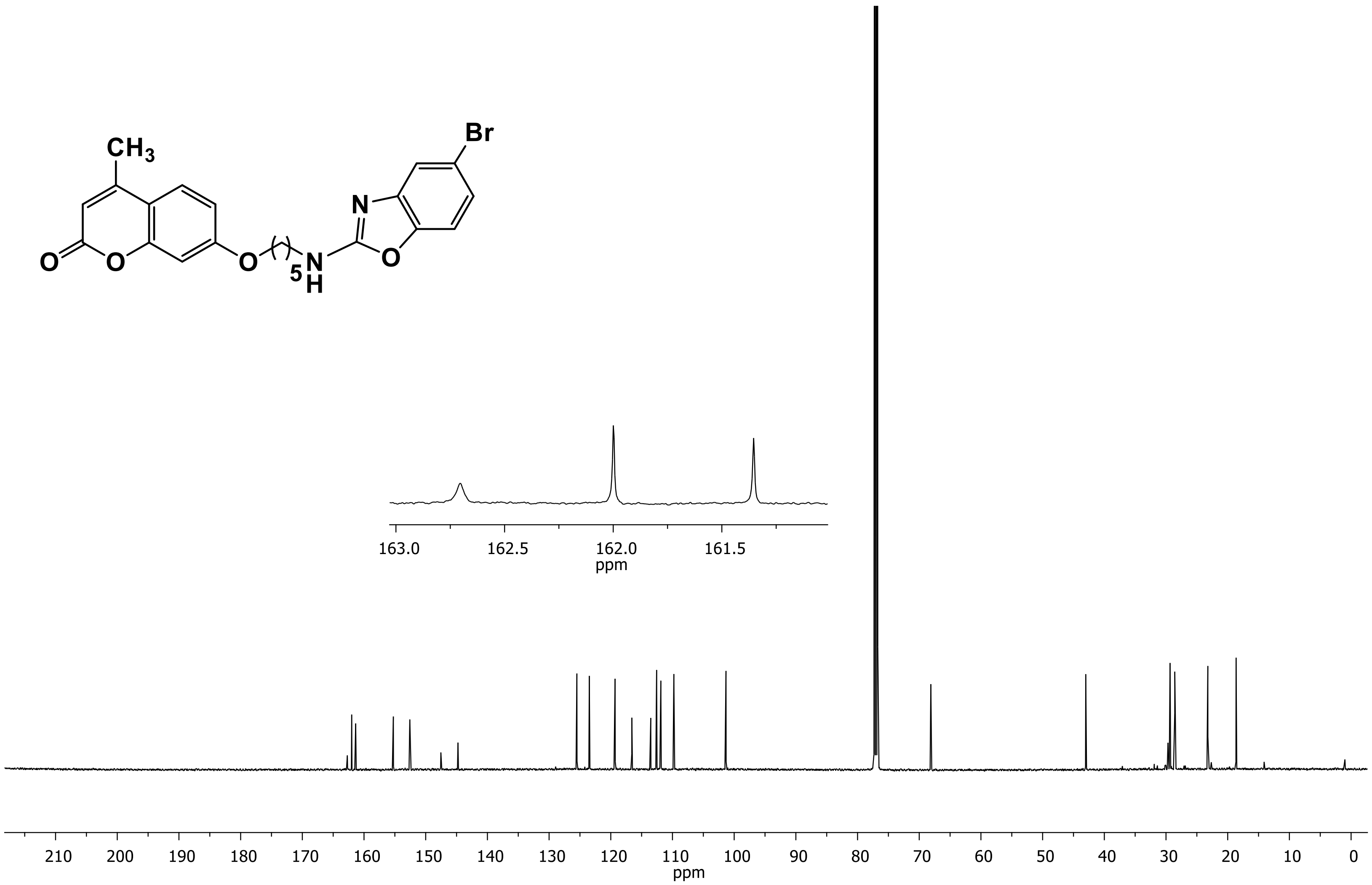

^{13}C-NMR (125.7 MHz, $CDCl_3$) of **8h**

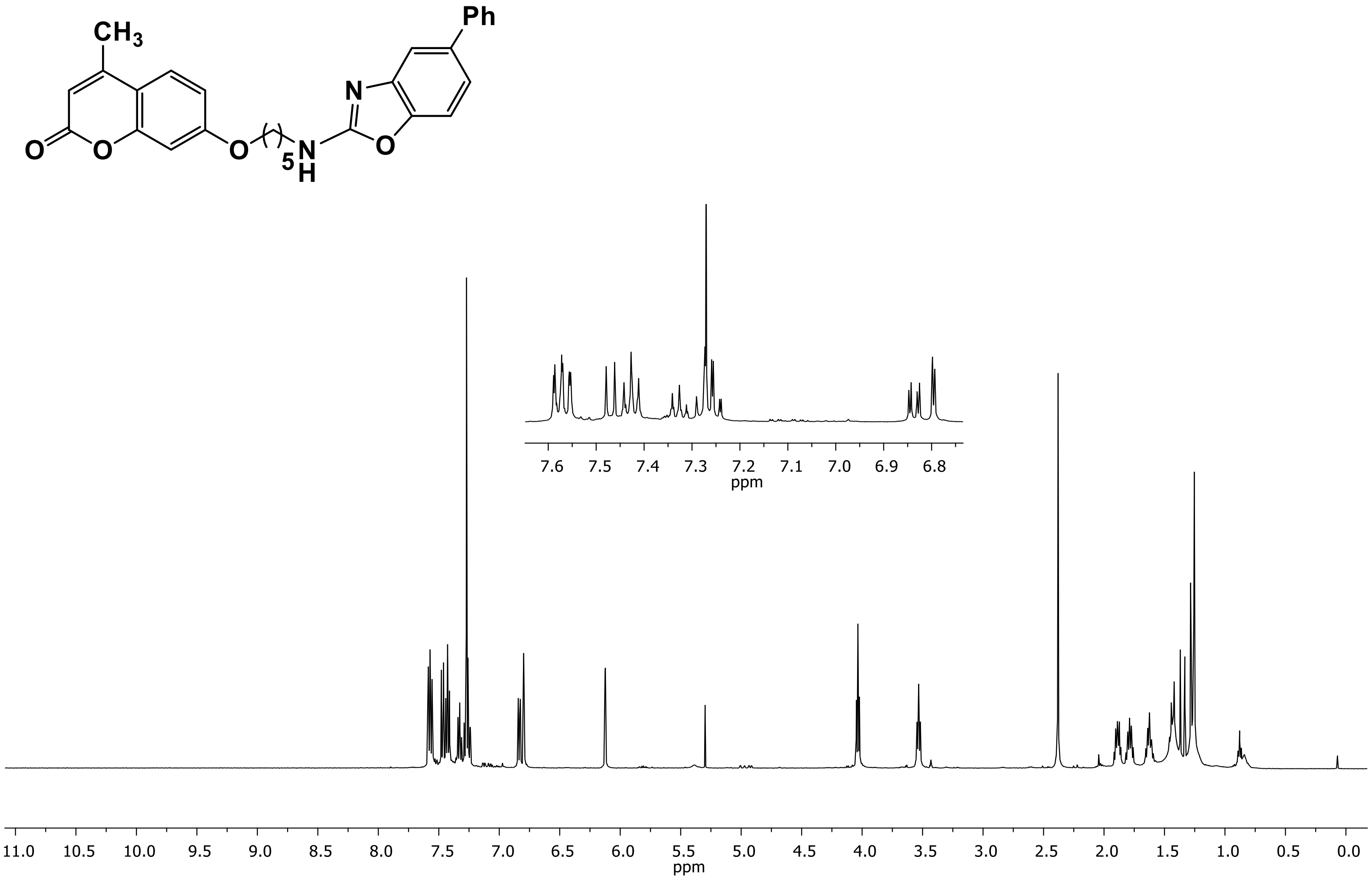

^{1}H-NMR (300 MHz, $CDCl_3/CD_3OD$) of **8i**

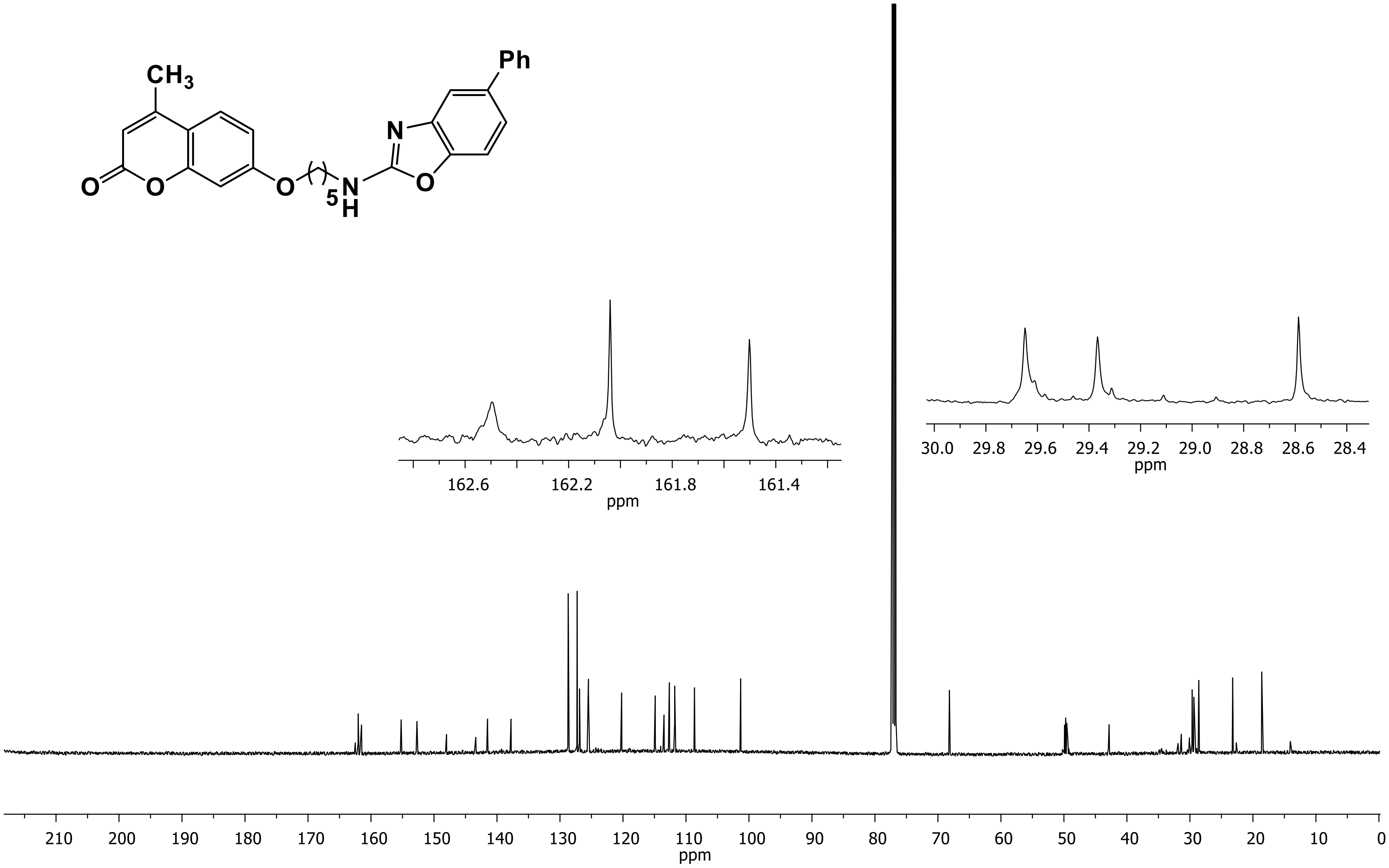

^{13}C-NMR (125.7 MHz, $CDCl_3/CD_3OD$) of **8i**

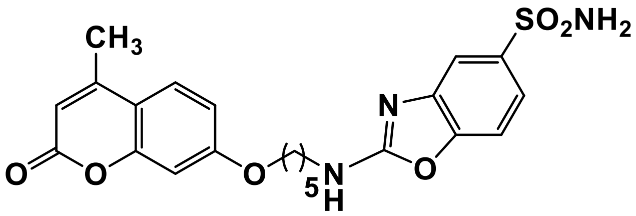

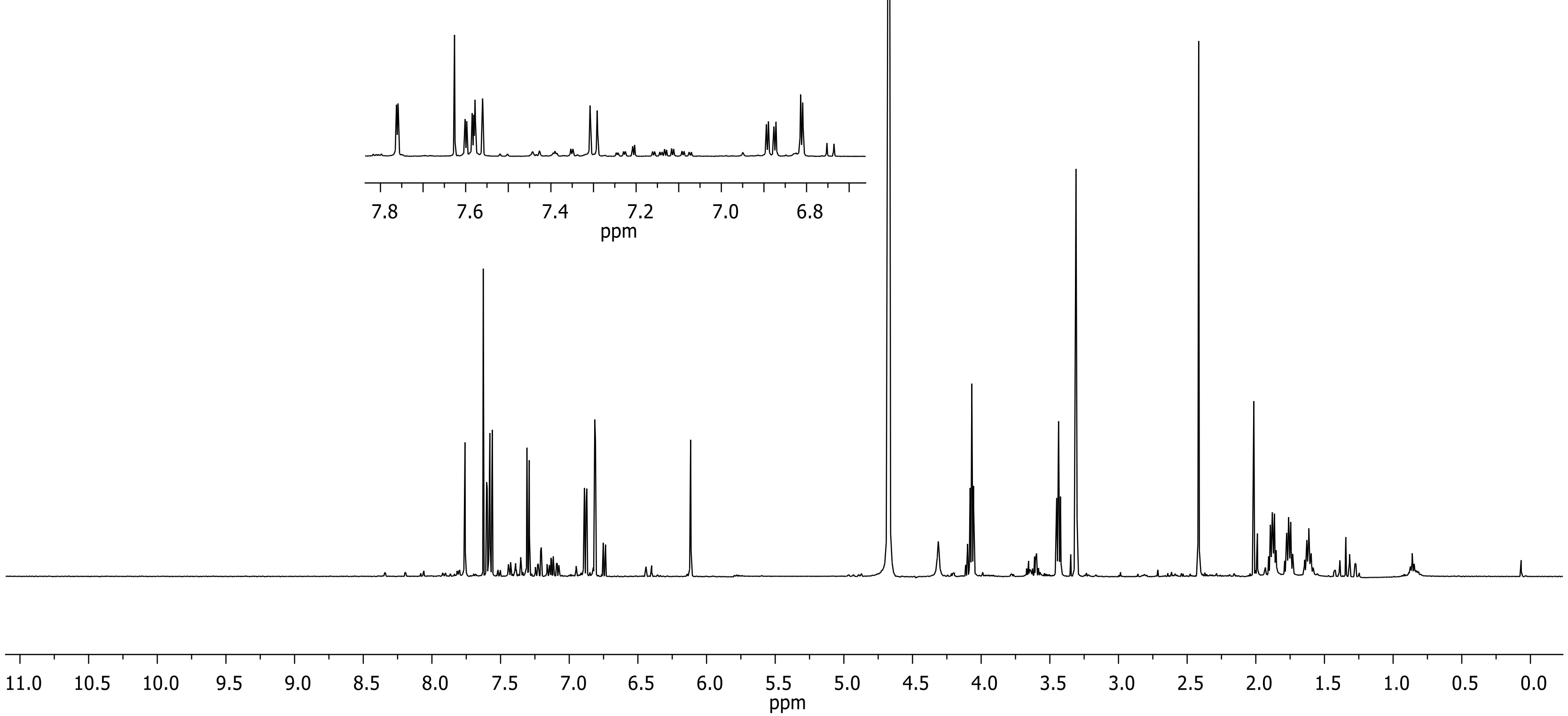

^{1}H-NMR (300 MHz, $CD_3OD/CDCl_3$) of **8j**

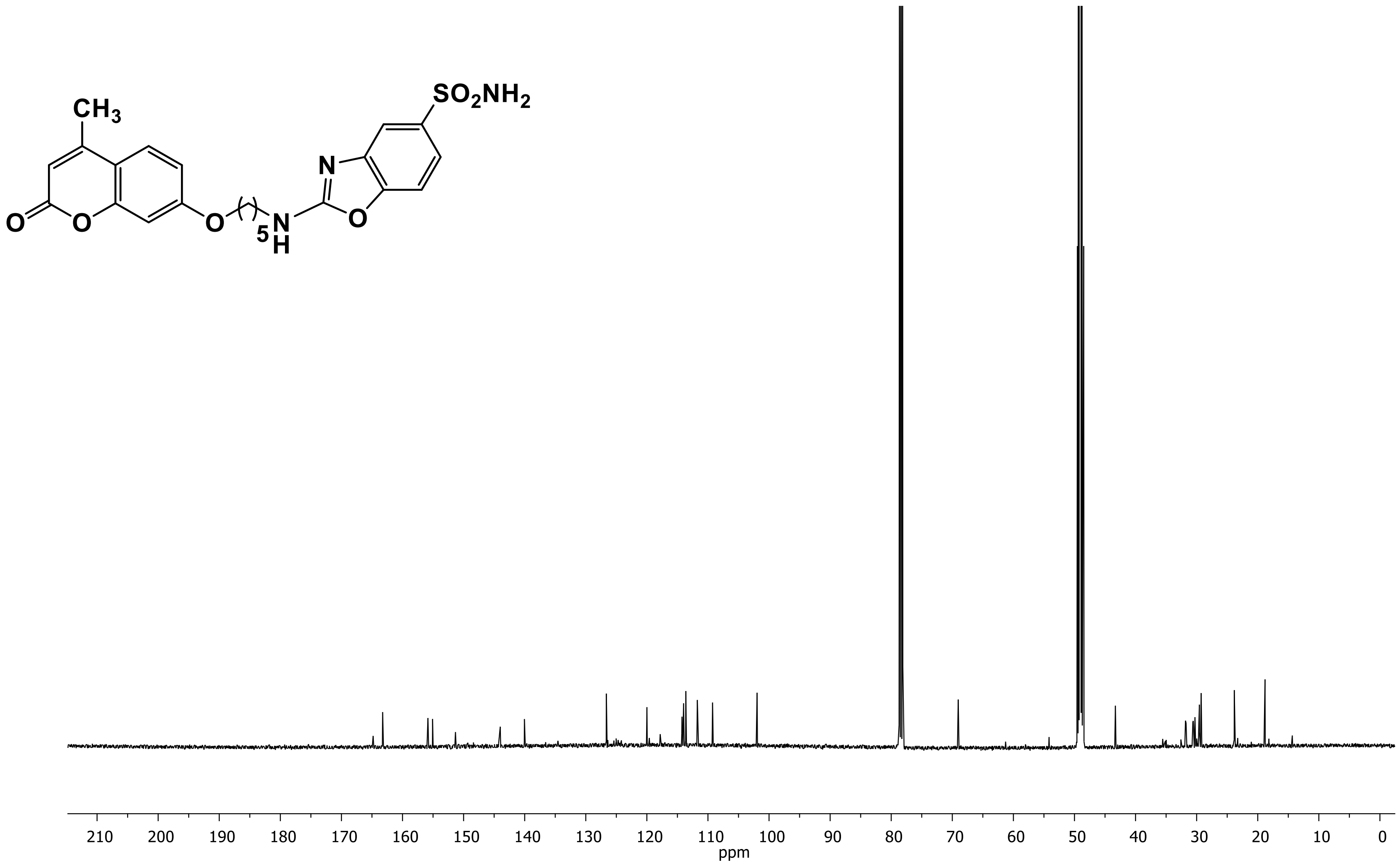

^{13}C-NMR (125.7 MHz, $CD_3OD/CDCl_3$) of **8j**
